# Morpheme Ordering Across Languages Reflects Optimization for Processing Efficiency

**DOI:** 10.1162/opmi_a_00051

**Published:** 2022-02-09

**Authors:** Michael Hahn, Rebecca Mathew, Judith Degen

**Affiliations:** Department of Linguistics, Stanford University; Department of Cognitive, Linguistic, and Psychological Sciences (CLPS), Brown University; SFB 1102, Saarland University

**Keywords:** language universals, morphology, information theory

## Abstract

The ordering of morphemes in a word displays well-documented regularities across languages. Previous work has explained these in terms of notions such as semantic scope, relevance, and productivity. Here, we test a recently formulated processing theory of the ordering of linguistic units, the efficient tradeoff hypothesis (Hahn et al., 2021). The claim of the theory is that morpheme ordering can partly be explained by the optimization of a tradeoff between memory and surprisal. This claim has received initial empirical support from two languages. In this work, we test this idea more extensively using data from four additional agglutinative languages with significant amounts of morphology, and by considering nouns in addition to verbs. We find that the efficient tradeoff hypothesis predicts ordering in most cases with high accuracy, and accounts for cross-linguistic regularities in noun and verb inflection. Our work adds to a growing body of work suggesting that many ordering properties of language arise from a pressure for efficient language processing.

## INTRODUCTION

Human language encodes thoughts into linear strings of words. Across languages, words are composed of morphemes, commonly defined as the smallest meaning-bearing units of language (Bloomfield, 1926; de Courtenay, 1972; Katamba, 2006). For instance, the English word “runners” can be decomposed into three morphemes: the root *run-* indicating an action, the suffix *-er-* indicating someone performing an action, and plural *-s* indicating a group of several referents. The ordering of morphemes within a word follows well-documented cross-linguistic tendencies (Baker, 1985; Bybee, 1985; Greenberg, 1963). For instance, derivational morphemes (e.g., English *-er* deriving nouns from verbs) are ordered closer to the root than inflectional morphemes (e.g., English plural *-s*). In morphologically rich languages, nouns and verbs often have a string of two or more affix morphemes attached to a root, and the typological literature has documented universal tendencies, such as a preference for plural markers to be closer to noun stems than case markers (Bybee, 1985; Greenberg, 1963).

Explaining these linguistic universals has been an important subject of study (Bauer, 2010; Bybee, 1985; Hay & Plag, 2004; Manova & Aronoff, 2010; Rice, 2011; Spencer, 2006). Explanations of morpheme ordering have been stated in terms of correspondences between morpheme ordering and morpheme meanings (Bybee, 1985; Rice, 2000; Saldana et al., 2021), parallelism between morphology and syntax (Baker, 1985; Givón, 1971; Vennemann, 1973), and human morphological processing and usage frequencies (Hay, 2002; Inkelas, 2016; Plag, 2002). Explanations of the first kind state that morphemes are ordered based on differences in semantic scope (Rice, 2000) or relevance (Bybee, 1985), such that morphemes that are semantically closer to the root occur closer to it in linear order. Explanations of the second kind propose that the ordering of morphemes mirrors the order of independent words with corresponding meanings, due to either language history or synchronic constraints on language. Explanations of the third kind argue that affixes are closer to the root when they are more likely to be processed together with it in dual-route models of lexical access (Baayen, 1993), which happens, for instance, when they are less productive.

A recent theory proposes a cognitive explanation for word and morpheme order in language, arguing that ordering universals in language optimize processing effort under memory limitations (Hahn et al., 2021). Hahn et al. (2021) introduce the notion of a *memory-surprisal tradeoff*: The more memory resources a comprehender invests in representing the context preceding the currently observed word, the lower the achievable surprisal that the comprehender must incur on that word. Conversely, the less memory is invested, the higher the surprisal. Hahn et al. (2021) argue for the *efficient tradeoff hypothesis*, the idea that the order of words and morphemes in language provides particularly efficient memory-surprisal tradeoffs. They show that optimizing the memory-surprisal tradeoff amounts to placing elements close together that strongly predict each other, as measured by mutual information. Hahn et al. (2021) argue that this property of the memory-surprisal tradeoff generalizes previous processing theories that suggest that orderings tend to place together elements that are syntactically related (Hawkins, 1994; Rijkhoff, 1986), conceptually related (Givón, 1985), semantically relevant to each other (Bybee, 1985), or processed together in lexical access (Hay & Plag, 2004). While focused on explaining word order across 54 languages, Hahn et al. (2021) also test whether two languages optimize the memory-surprisal tradeoff at the morphological level. In particular, they find that optimizing the memory-surprisal tradeoff partly reproduces the ordering of morphemes in Japanese and Sesotho verbs.

Here, we test this theory on a broader basis, considering both a larger set of languages and extending coverage from verbs to nouns. We consider data from four agglutinative languages—that is, languages with rich morphology where words tend to have multiple morphemes that are mostly realized separately (Korean, Turkish, Hungarian, and Finnish)—in addition to the two languages already considered by Hahn et al. (2021) (Japanese and Sesotho). These languages have very substantial verbal inflection, and three of these languages (Turkish, Hungarian, Finnish) also have substantial noun inflection. We test both whether the memory-surprisal tradeoff accounts for universals of verb affix ordering documented by Bybee (1985), but also extend the scope of the analysis to nouns, where we test whether the theory accounts for Greenberg’s Universal 39 (Greenberg, 1963).

The choice of the languages is guided and constrained by three factors: the presence of rich agglutinative morphology, the availability of corpus data with morphological annotation, and diversity in language families. The six languages under consideration represent five language families (Korean, Japonic, Uralic, Turkic, Niger-Congo). Finnish and Hungarian both belong to the Uralic family, sharing a common ancestor about 5,000 years ago (Maurits et al., 2020). The other languages in the sample are not genetically related according to their generally accepted classification (Hammarström et al., 2021).

In the remainder of the article, we first review prominent morpheme ordering universals in noun and verb inflection and the efficient tradeoff hypothesis, before testing it against data from the six languages and discussing our findings and the relation between the efficient tradeoff hypothesis and previous accounts of morpheme ordering.

## MORPHEME ORDERING AND ORDERING UNIVERSALS

In this section, we introduce the phenomena we seek to explain: crosslinguistic tendencies in the ordering of affix morphemes in nouns and verbs. Languages apply affix morphemes to different classes of words, including both open word classes such as nouns, verbs, and adjectives, and closed word classes, in particular, pronouns. In this work, we focus on open word classes, as these have productive paradigms that apply to thousands of words in a language, including words that newly enter the language, whereas pronominal inflection is restricted to a small number of words, often with idiosyncratic and fossilized paradigms inherited from earlier stages of a language. Among open word classes, inflection commonly applies to verbs, nouns, and adjectives. When adjectives are inflected, they often pattern with either verbs—when they are used as predicates—or nouns—when they are used as attributes or independent nouns. We thus focus on nouns and verbs, treating adjectives together with one of the other classes depending on the language when appropriate (with verbs in Korean and Japanese; with nouns in Hungarian, Finnish, Turkish).

### Universals of Noun Affix Ordering

**Figure 1.**
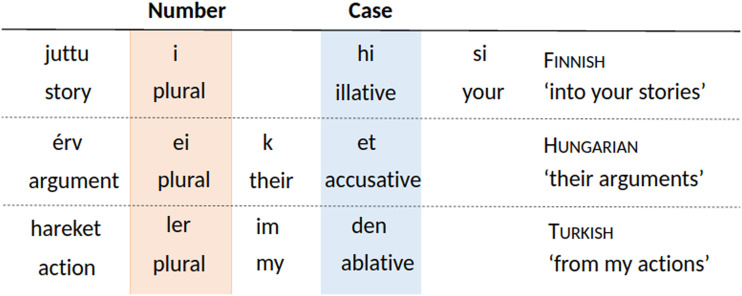
**Examples for noun inflection in the three languages in our sample.** All three languages support Greenberg’s Universal 39 by placing the case marker after the plural marker, but they differ in the placement of the possessive marker.

Nouns very commonly mark number and case morphologically (Dryer, 2013a, 2013c). In some languages, possession is also marked on the noun. Figure 1 shows fully inflected nouns in three languages from our sample, with endings for number, case, and possessor. Number and case marking are the subject of a well-documented universal, namely, Greenberg’s (1963) Universal 39:

Greenberg’s Universal 39: “the expression of number almost always comes between the noun base and the expression of case” (Greenberg, 1963, p. 112).

This universal is supported by the example in Figure 1.

### Universals of Verb Affix Ordering

Verb affixes are typically grouped into derivational and inflectional affixes. Derivational affixes derive new verb stems (e.g., “do” → undo), whereas inflectional affixes derive inflected verb forms from verb stems (e.g., “do” → ‘does’). Derivational affixes generally appear closer to the root than inflectional affixes (Greenberg, 1963).

The ordering of inflectional affixes shows universal tendencies (Bybee, 1985), which we summarize as follows:

Verb Affix Ordering (Bybee, 1985): Verb affixes are ordered as follows, outward from the verb stem:

verb stem valence voice TAM subject agreement

*Valence* affixes change the number of arguments. One type of valence affix is a causative, which adds an argument indicating who causes an event or state to occur (Song, 2013). *Voice* describes the distinction between active and passive. *Tense-Aspect-Mood* (*TAM*) comprises three types of categories (Tense-Aspect-Mood, Bybee et al., 1994; Dryer, 2013c). *Tense* describes where an event is located in time (e.g., past or future). *Aspect* describes how an event unfolds over time (Binnick, 1991; Comrie, 1976; Dahl, 1985). *Mood* describes a relation between an event and the speaker, including an assessment of the event’s reality (Palmer, 2001; Portner, 2018). One mood category is the potential mood, which indicates possibility. Aspect and tense categories are often fused in morphology (Binnick, 2012), and mood marking is also often fused with those. Some languages have a single affix slot that accommodates a fused morpheme indicating TAM. For instance, Finnish marks both tense (present and past) and mood (indicative, conditional, and potential) categories with a single morpheme. Other languages have multiple slots, for instance, Turkish TAM markers are distributed across three slots (see the Methods section). *Subject agreement* marks categories of the subject, most often its person and number, sometimes also other categories such as its gender (Corbett, 2003). Bybee (1985) also provides evidence for ordering preferences within aspect, tense, and mood; however, we do not distinguish between them as these are frequently fused in languages.

Having introduced the two universal generalizations about noun and verb affixes, we now review existing accounts of morpheme ordering.

### Previous Accounts of Morpheme Ordering

Here, we review previous explanatory accounts of morpheme ordering and motivate our study. Prominent accounts of morpheme ordering universals highlight the correspondence between morpheme ordering and semantics (Bybee, 1985; Rice, 2000). Bybee (1985) argues that ordering is determined by the semantic relevance of affixes to the root. For example, she argues that morphemes that change a verb’s argument structure, such as passives and causatives, have a particularly strong relation to the verb’s semantics, as they fundamentally alter the nature of the event described, whereas tense or agreement markers are much less tightly linked to the verb’s meaning. Similarly, she argues that agreement markers are less relevant to the stem than TAM markers, since TAM interacts more closely with the verb’s semantics; for instance, verbs denoting states or actions differ in the applicable aspect categories, but not in the applicable subject agreement features. While Bybee (1985) focused on verbs, arguably a similar argument can possibly be made for Greenberg’s Universal 39: A plural affix changes the referent of the noun from an individual to a group, whereas a case affix only describes the noun’s syntactic relation to the rest of the sentence.

The intuitive notion of relevance provides an appealing account of the Verb Affix Ordering generalization and Greenberg’s Universal 39. However, applying it to novel languages as an explanatory and testable notion requires some kind of formal operationalization of relevance that also applies to other language-specific kinds of morphemes, such as negation and politeness.

A second prominent semantic account holds that morphemes are ordered in the order in which their meanings combine, so that morphemes are closer to the root when their meanings have narrower scope (e.g., Caballero, 2010; Korotkova & Lander, 2010; Narrog, 2010; Rice, 2000). A good example for the scope-based explanation is the relative ordering of valence and voice. Turkish has suffixes both for causative and passive. When adding both suffixes simultaneously, the causative marker appears closer to the root. The Turkish verb stem *don* “to freeze” forms a causative *don-dur* “to freeze (something).”

Further applying a passive suffix results in *don-dur-ul* “to be frozen” (van Schaaik, 2020, section 30.8.2). The order of affixes corresponds to the order in which the meanings of these suffixes combine with the meaning of the root: The causative affix adds an argument indicating who makes an object freeze, and the passive affix then backgrounds that argument, yielding a verb describing something that is being frozen by someone.

This account is highly successful at predicting the order of valence and voice, among other morphemes (e.g., Caballero, 2010; Korotkova & Lander, 2010; Narrog, 2010; Rice, 2000), with the exception of anti-scope orderings in some languages (Hyman, 2003). However, it is not always straightforward to evaluate for other affixes, because its predictions depend on the specifics of how meaning is represented formally. For instance, there are cases where semantically equivalent affixes are ordered differently in different languages, for example, possessive suffixes are ordered differently in Finnish nouns than in Hungarian and Turkish nouns, seemingly without a motivating difference in semantic scope; the scope-based theory makes no prediction about how a given language’s affixes are ordered in such cases. Furthermore, there are scope-bearing items whose order varies between languages without apparent difference in meaning, for instance, negation appears closer to the root than TAM in Turkish and farther from it in Sesotho.

Relatedly, Saldana et al. (2021) argue that Greenberg’s Universal 39 reflects a cognitive bias favoring orderings that match conceptual structure. In an artificial language learning paradigm, they exposed participants to stimuli where nouns had a case or number marker (but not both), and then had participants extrapolate to forms containing both types of affixes. Learners of an artificial language strongly preferred the ordering described in Greenberg’s Universal 39, which Saldana et al. (2021) interpret as evidence for a cognitive bias favoring a match between linear ordering and conceptual structure. They also found that this preference could be reversed by making the form of the affix strongly dependent on the stem, which is not accounted for by conceptual structure, and which they interpret as reflecting a bias toward locality in dependencies.

Another family of theories hold that morpheme ordering mirrors the ordering of words (Baker, 1985; Givón, 1971; Vennemann, 1973). Under one kind of explanation, the ordering of morphemes reflects the ordering of formerly independent words that have been fossilized into bound morphemes, which can often be verified in languages where historical data is available (Givón, 1971; Vennemann, 1973). On the other hand, Bybee (1985) points out that there are historically documented cases where morpheme ordering has been restructured in ways that do not reflect former independent words, but respect the universal tendencies documented in the Morpheme Ordering and Ordering Universals section (see also Haspelmath, 1993; Mithun, 1994, 2000; Rice, 2000, section 15). This can happen both when affixes are reanalyzed (Bybee, 1985, p. 39) or when they change their meaning (Rice, 2000, section 15.1.3). A related proposal postulates a correspondence between the ordering of words and morphemes on a purely synchronic basis as a constraint on possible human languages. Baker (1985) proposed the Mirror Principle, which—informally—states that the ordering of elements (morphemes) in morphology reflects the ordering of elements (words) in syntax. However, this principle alone does not directly explain why elements are ordered the way they are in syntax and morphology. Unlike the other proposals discussed here, it also does not directly apply to the observed linear order of words and morphemes, but rather to a hypothetical underlying order before the application of movement operations assumed in certain theories of syntax.

A prominent cognitively motivated theory of morpheme ordering is the theory of complexity-based ordering (Hay, 2002; Hay & Baayen, 2005; Hay & Plag, 2004; Plag, 2002; Plag & Baayen, 2009). This theory holds that affixes are closer to the root when they are more likely to be processed together with the base in the dual-route model of human lexical access (Baayen, 1993). For instance, this model argues that more productive affixes are more likely to be accessed separately from the root than less productive affixes (Baayen, 1993). This theory has been applied to the ordering of derivational affixes in English, but not to the affix ordering generalizations described in the sections Universals of Noun Affix Ordering–Universals of Verb Affix Ordering. Relatedly, Inkelas (2016) proposed that morphemes are ordered together when they are informative about each other, using a notion of informativity introduced by Cohen Priva (2012). In a pilot study of Turkish, they found preliminary evidence that high-informativity suffixes are closer to the root.

Taken together, previous accounts explain the ordering of morphemes in terms of their meanings, their historical origins, or the way they are processed. These accounts all have independent merit and gaps in explaining morpheme ordering, accounting for complementary aspects of morpheme ordering by appealing to semantics, syntax, and human processing. We will now turn to the hypothesis that the generalizations arise from optimization for efficient memory-surprisal tradeoffs.

## LOCALITY AND THE MEMORY–SURPRISAL TRADEOFF

Here, we review the memory-surprisal tradeoff and a resulting hypothesis about the ordering of linguistic elements, the efficient tradeoff hypothesis, as an explanatory principle of ordering in language. We then test the efficient tradeoff hypothesis on morpheme ordering.

A long line of work in linguistics has proposed principles of locality to account for word ordering regularities within and across languages. In word order, the head adjacency or head proximity principles of Frazier (1985) and Rijkhoff (1986) state that words are close to their syntactic heads, a generalization that has found strong empirical support from data in many languages (e.g., Futrell et al., 2015; Hawkins, 1994; Liu, 2008; Liu et al., 2017). Explanations of these principles suggest that placing syntactically related words closer together makes human syntactic parsing more efficient and less sensitive to limitations in human memory (Frazier, 1985; Futrell et al., 2020; Gibson, 1998; Hawkins, 2003). Another group of theories holds that elements are closer together in linear ordering when they are semantically closer together in their meaning because this makes linear ordering iconically reflect relations between meanings (Givón, 1985). In morpheme ordering, Bybee (1985) argues that morphemes are closer to the root when they are more relevant to it; Hay (2002) and Plag (2002) argue that morphemes are closer to the root when they are more likely to be processed together with the root in human lexical access.

Hahn et al. (2021) proposed a cognitive principle that aims to unify and formalize these locality principles in the form of a memory–surprisal tradeoff. This is a cognitive account of the ordering of words and morphemes in human language, based on a formalization of memory efficiency in incremental processing. The memory-surprisal tradeoff links information-theoretic models of memory limitations with surprisal theory.

Surprisal theory (Hale, 2001; Levy, 2008) is a theory of the word-by-word processing difficulty in online processing. It states that the processing effort on a word *w*_*t*_ in context *w*_1_ … *w*_*t*−1_ is proportional to its surprisal

$$\text{Difficulty}\propto -\log_{2}P\left(w_{t}|w_{1}\ldots w_{t-1}\right),$$ (1)

where log_2_ denotes logarithms with base 2. Surprisal as estimated by corpus-based methods or cloze tasks is a successful predictor of reading time on naturalistic text (Aurnhammer & Frank, 2019; Frank & Hoeks, 2019; Goodkind & Bicknell, 2018; Smith & Levy, 2013; Wilcox et al., 2020). Surprisal theory is a computational-level theory (Marr, 1982); it can be implemented via different mechanisms, including preactivation and integration (Kuperberg & Jaeger, 2016). Futrell et al. (2020) and Hahn et al. (2021) argue that, due to limitations in human memory, human expectations in reality do not reflect the true context *w*_1_ … *w*_*t*−1_, but some potentially lossy memory representation *m*_*t*_ of the context *w*_1_ … *w*_*t*−1_:

$$\text{Difficulty}\propto -\log_{2}P\left(w_{t}|m_{t}\right).$$ (2)

Hahn et al. (2021) note that there is a tradeoff between average surprisal and memory capacity: The more information a listener stores in *m*_*t*_, the lower their surprisal will be on average. This is because higher precision of memory leads to more precise expectations, which will achieve lower surprisal on average.

More formally, they consider functions *M* describing how comprehenders update memory representations *m*_*t*−1_ when observing a word (or morpheme) *w*_*t*_ and integrating it into a new memory state *m*_*t*_ := *M*(*m*_*t*−1_, *w*_*t*_). The memory capacity is formalized as the average number of bits required to encode *m*_*t*_, that is, its entropy:

$$H\left[m_{t}\right]≔-\sum_{m}P\left(m_{t}=m\right)\log_{2}P\left(m_{t}=m\right),$$

where *m* runs over possible memory states. Hahn et al. (2021) prove that there is a tradeoff between the average surprisal *S*_*M*_ obtained by averaging −log *P*(*w*_*t*_, *m*_*t*_) across the words in a text, and the memory capacity H[*m*_*t*_].

**Figure 2.**
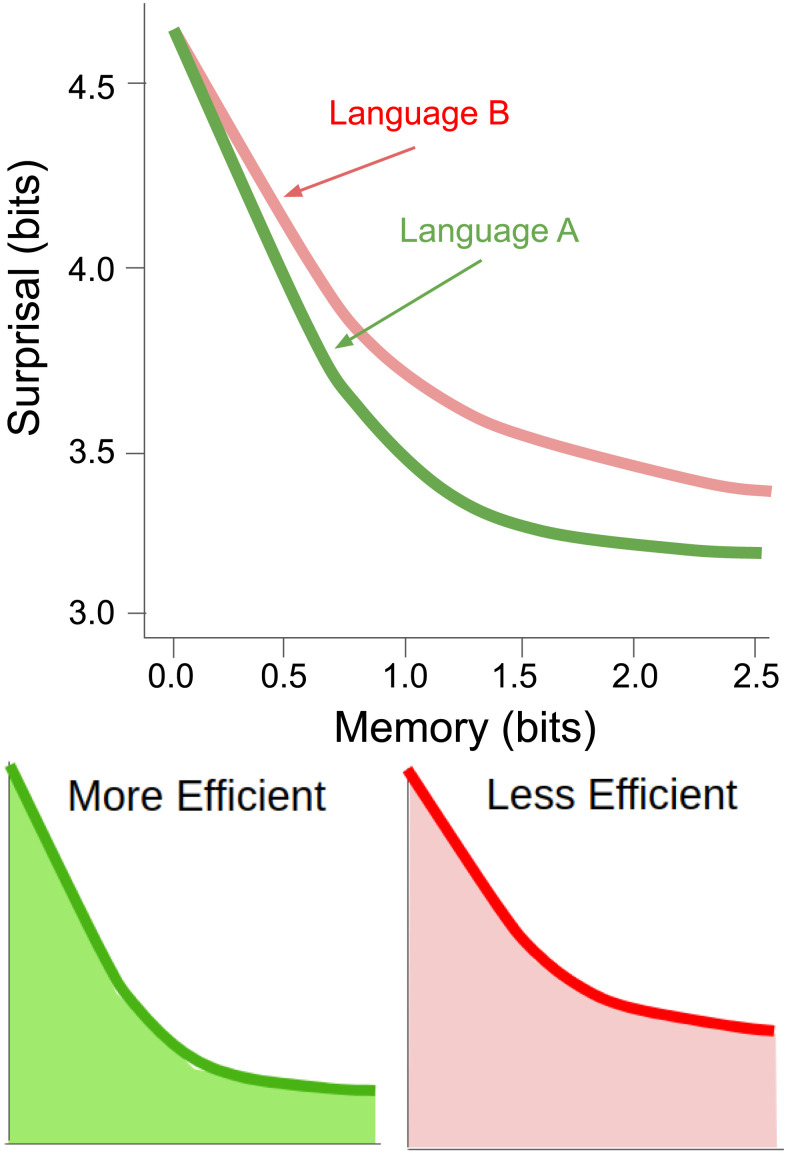
**Memory-surprisal tradeoff curves.** *Top*: The memory-surprisal tradeoff in two hypothetical languages: In order to achieve a given level of surprisal, a comprehender has to invest a certain amount of memory resources, which can be quantified information-theoretically in terms of bits. In this case, Language A provides a more efficient tradeoff because comprehenders can achieve lower surprisal than Language B with the same memory load. *Bottom*: The area under the curve (AUC) for the two hypothetical languages. Language A has a lower AUC than Language B, corresponding to a more efficient memory-surprisal tradeoff.

Different orderings can lead to different tradeoffs that in turn can differ in their efficiency (Figure 2): Tradeoffs are more efficient when comprehenders can achieve lower surprisal for the same amount of memory. The efficiency of a tradeoff curve can be quantified using its area under the curve (AUC) (Hahn et al., 2021): There is a smaller area under a more efficient tradeoff curve, such as that of Language A in Figure 2. Hahn et al. (2021) propose the efficient tradeoff hypothesis: Human language orders elements in such a way that the memory-surprisal tradeoff is particularly efficient, compared to other possible orderings.

To test this hypothesis, Hahn et al. (2021) provide a method for estimating the memory-surprisal tradeoff from corpus data. This method is based on the notion of mutual information (Cover & Thomas, 2006), which quantifies the amount of statistical association between two random variables. If *X*, *Z*, *Y* are random variables, then the mutual information of *X* and *Y*, conditioned on *Z*, is defined to be:

$$I\left[X:Y|Z\right]\equiv \sum_{x,y,z}P\left(x,y,z\right)\log_{2}\frac{P\left(x,y,z\right)}{P\left(x,z\right)P\left(y,z\right)}.$$ (3)

The mutual information I[*X* : *Y*|*Z*] quantifies how much predictive information *X* and *Y* provide about each other, assuming one already has access to the covariate *Z*. The key quantity derived from this is the mutual information between elements (such as morphemes) that are at some distance *t*, conditioned on the intervening elements:

$$I_{t}\equiv I\left[w_{t}:w_{0}|w_{1},\ldots ,w_{t-1}\right].$$ (4)

In the definition of mutual information Equation 3, *w*_*t*_ and *w*_0_ corresponds to *X* and *Y*, respectively, whereas *Z* corresponds to the string *w*_1_ … *w*_*t*−1_. Thus,

$$I_{t}\equiv \sum_{w_{0}\ldots w_{t}}P\left(w_{0}\ldots w_{t}\right)\log_{2}\frac{P\left(w_{0}\ldots w_{t}\right)}{P\left(w_{0}\ldots w_{t-1}\right)P\left(w_{1}\ldots w_{t}\right)}.$$ (5)

Based on this notion, Hahn et al. (2021) prove a bound on the memory-surprisal tradeoff: Assume that a comprehender’s memory capacity is bounded as follows, for some positive integer *T*:

$$H\left[m_{t}\right]\leq \sum_{t=1}^{T}{tI}_{t}.$$ (6)

Informally this quantity measures the amount of information that would need to be stored to capture predictive information from *T* preceding words, where each bit of information is weighted by the distance over which it has to be remembered (and thus occupies memory resources). Then there is a lower bound on the average surprisal *S*_*M*_ experienced by that comprehender:

$$S_{M}\geq S_{\infty }+\sum_{t=T+1}^{\infty }I_{t}.$$ (7)

where *S*_∞_ is the average surprisal that would be achieved with perfectly veridical memory representations. Informally, the sum on the right side describes information between words at a distance of more than *T*. This information cannot be captured when memory is bounded as in Equation 6. Psycholinguistic research has proposed a wide range of theories of the content and architecture of memory states (e.g., Gibson, 1998; Just & Carpenter, 1992; Lewis & Vasishth, 2005; MacDonald & Christiansen, 2002; McElree et al., 2003). Remarkably, even though the quantities in Equation 6 and Equation 7 are defined in terms of sequences of adjacent words, Hahn et al. (2021) prove this bound independently of any assumption about what information is stored by the memory encoding function *M*.

Because *I*_*t*_ can be estimated from text data, this result yields a method for estimating a bound on the tradeoff curve from text data by tracing out possible memory capacities H[*m*_*t*_] from 0 to +∞.

Hahn et al. (2021) show that tradeoffs are more efficient when pairs of elements with higher mutual information are ordered close together, a property they refer to as information locality. Expressed in terms of mutual information, information locality corresponds to a steep fall-off of *I*_*t*_ as *t* increases. This means that predictive information about a word tends to be concentrated in the recent past. Information locality optimizes the memory-surprisal tradeoff because it reduces the need to accumulate information over long sequences of words, and enables contextual information to be brought to bear on processing new words soon after it is encountered. Formally, information locality is implied by the factor *t* inside the sum in the memory bound in Equation 6: It states that memory cost is impacted more strongly by *I*_*t*_ when the distance *t* is larger.

Hahn et al. (2021) argue that this information-theoretic notion of locality derives a range of locality principles proposed in the linguistic literature, such as the idea that syntactically related words tend to be close in linear distance (Ferrer i Cancho, 2004; Futrell et al., 2015; Hawkins, 1994; Liu, 2008; Liu et al., 2017; Rijkhoff, 1986; Temperley & Gildea, 2018). Beyond providing evidence that word orders provide efficient tradeoffs, they also provide preliminary evidence that it accounts for some properties of morpheme ordering, using data of verb inflection in two languages (Japanese and Sesotho).

In this work, we aim to test the efficient tradeoff hypothesis as a predictor of morpheme ordering more broadly, using data from more languages and from different parts of speech. That is, we test whether morpheme ordering is more efficient than most other possible ways of ordering morphemes, and whether this accounts for the universal tendencies documented in the sections Universals of Noun Affix Ordering–Universals of Verb Affix Ordering.

We discuss connections between the efficient tradeoff hypothesis and previous theories of morpheme ordering in the section Relation to Previous Accounts.

## TESTING THE EFFICIENT TRADEOFF HYPOTHESIS

We test the efficient tradeoff hypothesis as a predictor of morpheme ordering. To this end, we evaluate whether real orderings of morphemes lead to more efficient tradeoffs than most other possible orderings, and, whether the properties of real orderings arise from optimizing for the tradeoff’s efficiency.

### Methods

#### Data.

We selected data from languages that have rich agglutinative morphology, that is, languages in which (i) verbs and nouns often have more than two morphemes per words, as that allows us to test predictions about the relative ordering of different morphemes, and (ii) the morphemes within a word have clearly delimited boundaries, providing unambiguous information about the ordering of morphemes. Beyond the languages studied in Hahn et al. (2021), we obtained data from four such languages from Universal Dependencies (UD; Nivre et al., 2020) 2.6: Korean (Chun et al., 2018), Turkish (Coltekin et al., 2020), Hungarian (Farkas et al., 2020), and Finnish (Ginter et al., 2020; Piitulainen & Nurmi, 2020). In addition, we also reanalyze the data from Hahn et al. (2021), covering UD data for Japanese (Asahara et al., 2018) and the Child Language Data Exchange System (CHILDES) Sesotho corpus (Demuth, 1992) in a way consistent with our analysis of the other four languages. We obtained between 7,328 (Hungarian) and 65,541 (Finnish) inflected noun tokens and between 2,735 (Hungarian) and 109,323 (Korean) inflected verb tokens in each language. There were between 4,882 (Hungarian) and 47,846 (Finnish) distinct inflected noun types, and between 1,814 (Hungarian) and 30,818 (Korean) inflected verb types per language.

In the noun analyses we focused on Turkish, Hungarian, and Finnish, as nouns in these languages often have more than one affix. In the verb analyses we used all six languages. For each language, we selected nouns and verbs based on the part-of-speech annotation in each corpus. We treated adjectives together with nouns in Hungarian, Finnish, and Turkish, and together with verbs in Korean and Japanese. We used available corpus annotation together with the grammatical literature on each language to determine which morphemes each extracted word was composed of (see the Supplemental Materials Appendix, Section S1 for details).

**Figure 3.**
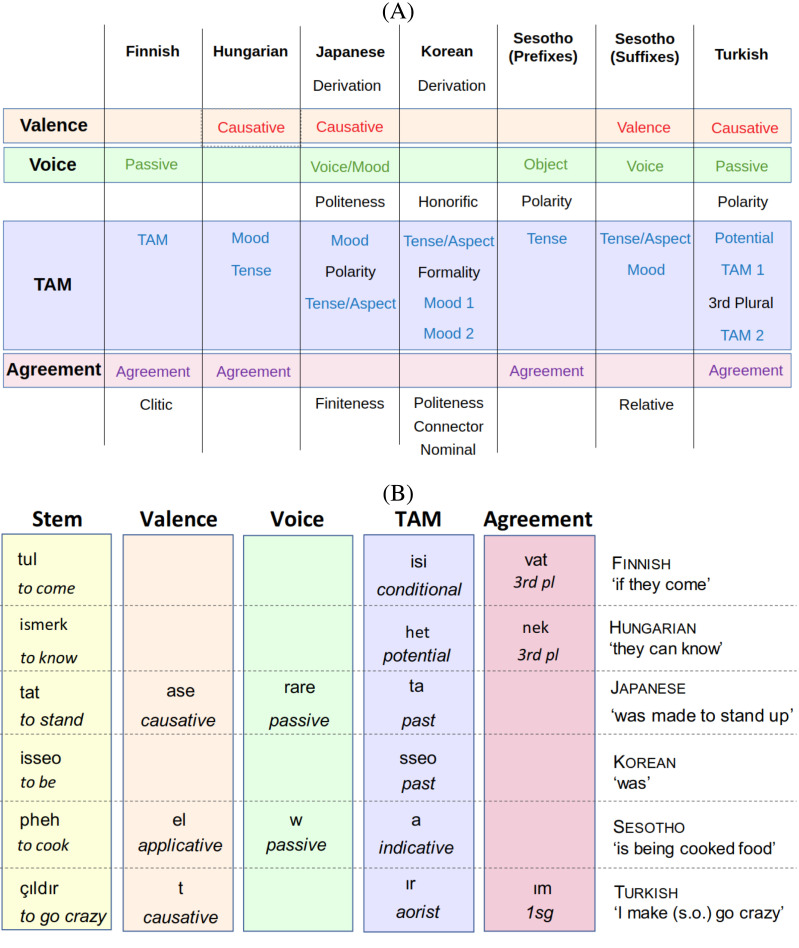
**Verb affixes in the languages from our sample.** (A) Verb affix slots in the six languages, grouped into four universal slots where applicable. Affixes are listed outwards from the root. (B) Examples of verb inflection from the six languages.

The languages in the sample generally support the two universals introduced in the section Morpheme Ordering and Ordering Universals. Figure 1 shows fully inflected nouns in three languages from our sample, with endings for number, case, and possessor. Figure 3 summarizes the affixes in the verbal morphologies of the six languages considered here (see the Supplemental Materials Section S1 for details on how we arrived at these summaries). Sesotho has both prefixes and suffixes; we treat these separately, as the universals under consideration here only concern the relative distance of affixes from the base, not which side of the verb they appear on. Figure 3 shows that the languages in our sample largely support the Verb Affix Ordering universal, with the exception of the ordering of the special third-plural suffix slot in Turkish, which intervenes between two TAM slots.

Examples of derivational suffixes are Japanese -su- and Korean -ha-, which derive verbs from nonverbal stems (Hasegawa, 2014; Yeon & Brown, 2010). Another example is the reversive suffix in Sesotho (corresponding to “un-” in English “do” → “undo,” Doke & Mofokeng, 1967).

Besides derivation and the four classes described in the universal, some further types of affixes occur in the six languages of our sample. While agreement is most commonly established with the subject (Dryer, 2013b), *agreement with the object* is found in Sesotho (Doke & Mofokeng, 1967) (in person and noun class) and in Hungarian (Rounds, 2001) (in definiteness). In Hungarian, it is fused with subject agreement and we treat the fused form as a single suffix. In Sesotho, it shares a slot with the reflexive voice affix (see the Supplemental Materials, Section S1), and we treat it as a voice affix because an object referenced by it is not realized by a noun phrase (Doke & Mofokeng, 1967, section 459). *Polarity* refers to the opposition between affirmative (e.g., “she arrived”) and negative (e.g., “she did not arrive”) statements (Dryer, 2013c). *Formality*, honorifics, and politeness are categories that index social relations between the speaker, the addressee, and the topic of the conversation (Hasegawa, 2014; Yeon & Brown, 2010). In our sample, these are prominent in Korean and Japanese. The Japanese politeness marker *-masu-* and the Korean formality (*-p*) and politeness (*-yo*) suffixes index the social relation between the speaker and the addressee (Hasegawa, 2014; Yeon & Brown, 2010); the Korean honorific suffix *-si-* indexes the social relation between the speaker and the topic of the conversation (Yeon & Brown, 2010). Furthermore, verb forms can have affixes indicating the syntactic position of the verb within a sentence, in particular, affixes marking infinitives or other nonfinite forms. Examples are the *Finiteness* slot (used to mark nonfinite verb forms) in Japanese, the *Connector* and *Nominal* slots (used to mark embedded and nominalized verbs) in Korean, and the *Relative* slot (used inside relative clauses) in Sesotho.

How might morphological properties affect mutual information? One key aspect is cooccurrence restrictions: Mutual information between two elements is higher when the presence of one constrains the presence of the other. For instance, in verbs, passive affixes can typically be only applied to certain verbs, in particular, transitives, whereas agreement affixes will typically be applicable to all verbs. In nouns, some nouns can only form a singular or only a plural (e.g., for English: Huddleston & Pullum, 2002, section 3.2), so that there is nonzero mutual information between the noun stem and the presence of a plural affix. In contrast, there may be no lexical restrictions on the case a noun appears in, as that only depends on the noun’s syntactic role in the sentence, potentially leading to lower mutual information between noun stems and case affixes. Besides hard grammatical constraints, statistical cooccurrence patterns grounded in semantics or usage patterns also impact mutual information. Number is again an example: Nouns may differ in their likelihood of occurrence in singular or plural number. For instance, “*shoe*” is much more likely to be used in the plural than “*hat*” in a large corpus of American English (Davies, 2012). Plausibly, all affix classes appearing in the universals have some degree of statistical cooccurence relation with the root: for instance, stative verbs might be less likely to take progressive aspect marking (Comrie, 1976, p. 36), and verbs like “to rain” are unlikely to take non-third-person subject agreement. Which orderings optimize information locality, and thus the memory-surprisal tradeoff’s efficiency, will depend on the relative strength of different cooccurrence relations in a language.

#### Applying the Efficient Tradeoff Hypothesis to Morpheme Ordering.

In order to estimate memory-surprisal tradeoffs, we model words as strings of morphemes, following Hahn et al. (2021). For instance, we represent Finnish *juttu-i-hi-si* “into your stories” (Figure 1) as juttu-Plural-Illative-2sgPoss. For each language, we parameterize possible morpheme orderings through the *N*! possible orderings of the *N* affix slots. Applying any such ordering to the forms extracted from the corpus results in a set of counterfactual forms with some associated memory-surprisal tradeoff curve. Following Hahn et al. (2021), we optimize orderings using an adaptation of the hill-climbing method originally devised by Gildea and Temperley (2007) for optimizing word order. See the Supplemental Materials, Section S3, for details on the estimation of mutual information, the memory-surprisal tradeoff curve, and the optimization method.

We compare the real orderings (*real*) to four different kinds of alternative orderings: First, we consider randomized morpheme orderings (*random*); these represent the set of all *N*! possible orderings of the *N* affix slots. Second, we consider random morpheme orderings that respect the universals discussed in the section Morpheme Ordering and Ordering Universals (*universals*).^1^ Third, we consider the reversed real orderings (*reverse*), and morpheme orderings that are optimized to minimize AUC under the tradeoff curve (*optimized*). We estimate memory-surprisal tradeoffs and computationally optimized orderings for the AUC under the tradeoff curve using the method described in Hahn et al. (2021).

If the efficient tradeoff hypothesis accounts for morpheme ordering, then we expect that real orderings are more efficient than most other possible orderings, and close to the most efficient possible orderings. We also expect that optimized orderings largely match the real orderings, to a higher degree than most other possible orderings. If the efficient tradeoff hypothesis predicts morpheme ordering even beyond the two universals, then real orderings should be more efficient even than most other orderings respecting the universals, and optimized orderings should resemble real orderings more than most other orderings respecting the universals.

### Results

**Figure 4.**
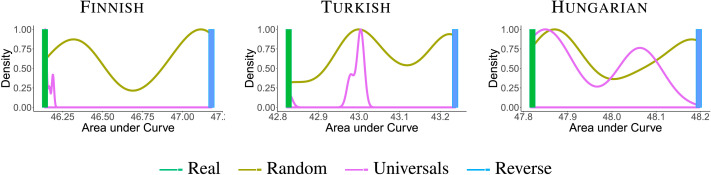
**AUC histograms for noun suffixes.** We show smoothed histograms of baseline orderings (brown) and orderings satisfying the universals (purple), and the AUC values for real (green) and reverse (blue) orderings. Lower AUC values indicate a more efficient tradeoff. Optimized orderings do not differ perceptibly from real orderings in AUC for nouns and are not plotted separately here.

**Figure 5.**
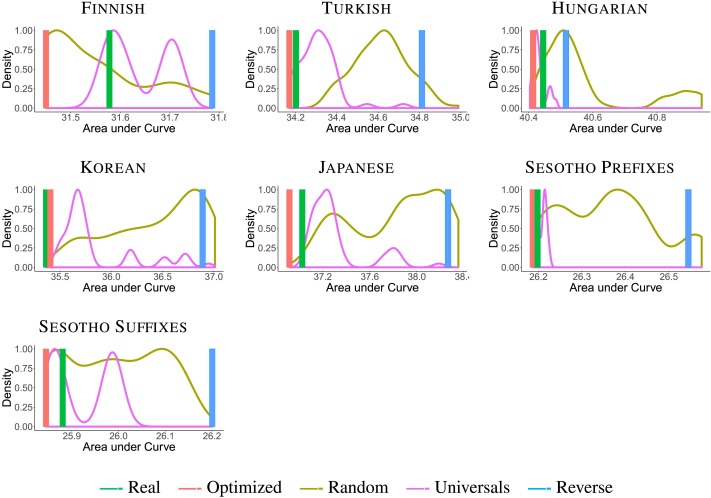
**Area under the curve (AUC) histograms for verb affixes.** We show smoothed histograms of baseline orderings (brown) and orderings satisfying the universals (purple), and the AUC values f or optimized (red), real (green), and reverse (blue) orderings. Lower AUC values indicate a more efficient tradeoff.

First, we evaluate the efficiency of real orderings compared to the baselines. Figures 4 and 5 show AUC plots for random orders as compared to the real ordering of morphemes for verbs and nouns, respectively. In most languages, real orderings have lower AUC than the vast majority of random baseline orderings, including the baselines that satisfy the universals. This is true for both nouns and verbs. This suggests that real morpheme orderings enable more efficient memory-surprisal tradeoffs than most of the *N*! possible orderings. Finnish verbs form the only exception; AUCs of real orderings are similar to those of baseline orderings (discussed later).

**Figure 6.**
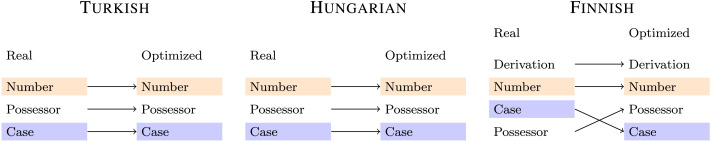
**Real and optimized ordering (nouns).** Colors indicate universal position slots relevant for Greenberg’s Universal 39.

**Figure 7.**
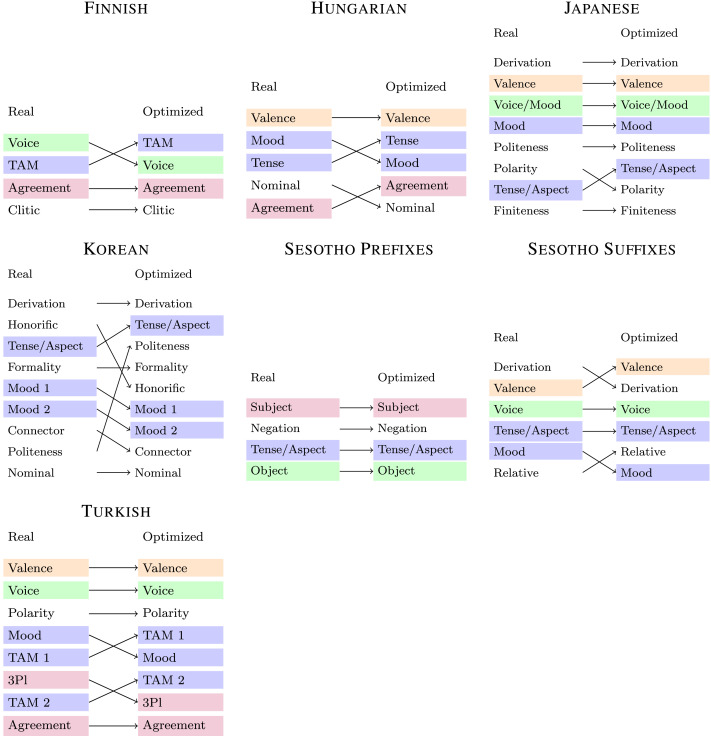
**Real and optimized ordering for verb affixes.** Colors indicate universal position slots as in Figure 3.

Second, we compare real and optimized orderings to evaluate whether optimization predicts the two universals. Figures 6 and 7 directly compare real and optimized orderings for nouns and verbs respectively. For the nouns, Greenberg’s Universal 39 is recovered by all optimized orderings. While there is a mismatch between real and optimized orderings in the language-specific ordering of possessive suffixes in Finnish, the AUC difference between real and optimized orderings is imperceptible in Figure 4. For the verbs, optimized orderings match the universal ordering for the morphemes occurring in each of these languages, except for Finnish verbs (discussed later). This includes one case (Turkish third-person plural agreement marker *-lar*) where the real ordering does not observe the universal, but the optimized ordering does.

Third, we evaluate the quantitative similarity between real and optimized orderings by measuring the accuracy of optimized orderings in predicting real orderings. If the efficient tradeoff hypothesis predicts morpheme order, then optimized orderings should achieve a higher prediction accuracy than most other possible orderings.

**Figure 8.**
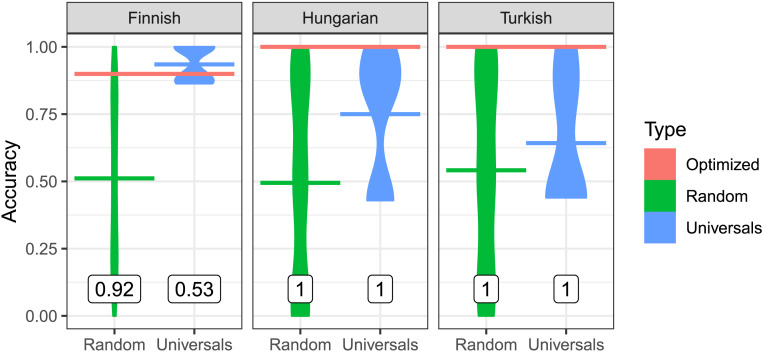
**Accuracies in predicting noun morpheme ordering.** The accuracy of optimized orderings is indicated by the red bars at the top. For the baselines, we provide both smoothed violin plots of the distribution of accuracies, and horizontal lines indicating mean accuracies. Random baselines have a mean accuracy of about 50%; baselines respecting the universals tend to have higher accuracies. The numbers indicate what fraction of baselines (random or universal-constrained) have a lower accuracy than the optimized ordering.

**Figure 9.**
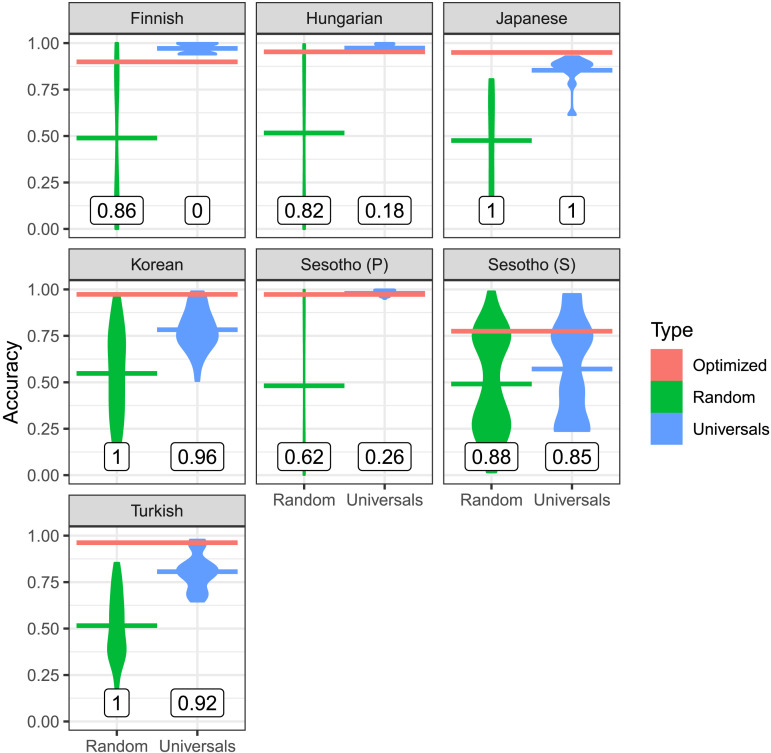
**Accuracies in predicting verb morpheme ordering.** For the baselines, we provide both a smoothed violin plot of the distribution of accuracies, and the mean accuracy as a horizontal line (green and blue). For the optimized order, we show the accuracy as a horizontal line (red). The numbers indicate what fraction of baselines (random or universal-constrained) have a lower accuracy than the optimized ordering. In all languages, optimized orderings provide higher accuracy than the majority of random baselines. In some languages, they additionally have higher accuracy than most universal-constrained baselines.

Figures 8 and 9 show the accuracy of optimized and random baseline orderings in predicting real orderings. We measured accuracy by counting what fraction of all pairs of affixes within a single word from the corpus are ordered in the same relative order as under the real ordering (see the Supplemental Materials Appendix, Section S2, for other ways of quantifying accuracy, with very similar results).

For nouns, the accuracy of optimized orderings is far above the agreement of most random grammars, outperforming 90% or more of random baseline orderings (Figure 8). For verbs, accuracy of optimized orderings is at least 90% in all cases except Sesotho suffixes. For Sesotho suffixes, optimized orderings still have an accuracy of 77%, higher than 88% of random baseline orderings. Taken together, across languages, morpheme ordering is predicted with high accuracy, consistently higher than what would be expected at chance.

We next compare to the universal-constrained baselines to evaluate whether optimization predicts ordering beyond the Verb Affix Ordering universal. In some languages (Finnish, Hungarian, Sesotho prefixes), the real ordering is already explained nearly entirely by this universal; here, optimized orderings do not outperform the universal-constrained orderings. However, in those languages where there are significant language-specific regularities going beyond the universal, so that universal-constrained baselines do not all achieve near-perfect accuracy (Japanese, Korean, Sesotho suffixes, Turkish), optimized orderings again consistently outperform most universal-constrained orderings. This suggests that the efficient tradeoff hypothesis accounts for some language-specific ordering patterns beyond those captured by the Verb Affix Ordering universal.

Finnish verbs are the only case where the optimized ordering does not seem to agree with the universal: Optimized orderings place the voice marker further from the root than the TAM marker, in disagreement with the real order. This can be traced to the properties of the Finnish form commonly called “passive”: The Finnish passive is marked by two morphemes, conventionally regarded as a voice marker (*-t-*) and an agreement marker (*-Vn*, Karlsson, 1999, section 69); we followed this convention in Figure 3. Functionally, these two morphemes always appear together and have no distinct meanings. Both can equally well be regarded as markers of the passive; there is no reason other than the match with the Verb Affix Ordering universal for the conventional view that one is a voice marker and the other is an agreement marker. More interestingly, unlike the passive of most languages, the Finnish passive is insensitive to the verb’s argument structure, simply denoting that an unspecified agent performed an action (Blevins, 2003; Shore, 1988). Therefore, the theories of semantic relevance and semantic scope discussed in the section Previous Accounts of Morpheme Ordering would arguably also predict the Finnish “passive” marker to pattern with agreement markers, unlike the actual Finnish ordering but in agreement with the Efficient Tradeoff Hypothesis. The Finnish “passive” may thus illustrate a language-specific idiosyncrasy not predicted by explanatory crosslinguistic accounts.

Finally, to elucidate the connection between ordering and mutual information, we computed the conditional mutual information (Equation 3) between affix classes and roots for nouns and verbs in the optimized orderings across languages. The conditional mutual information between the root and an affix class *C* indicates by how much surprisal of affixes in one class is reduced by knowledge of the root (or the reverse, in the case of prefixes), averaging across all words where affixes of both classes appear. That is, we consider all strings *w*_1_, … , *w*_*k*_ in the dataset where *w*_1_ is the root and *w*_*k*_ belongs to affix class *C*, and compute the pointwise conditional mutual information

$$\log_{2}\frac{P_{k}\left(w_{k}|w_{1}\ldots w_{k-1}\right)}{P_{k-1}\left(w_{k}|w_{2}\ldots w_{k-1}\right)},$$ (8)

and obtain the information between two affix classes by averaging over all such strings.

**Figure 10.**
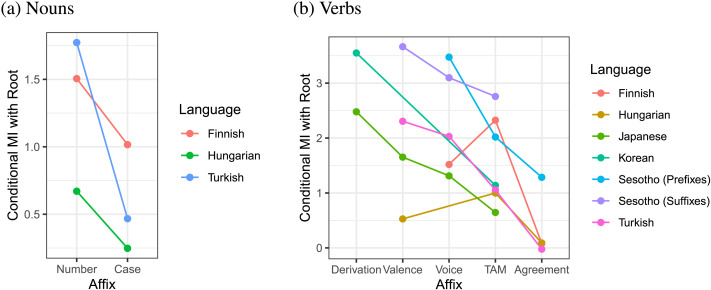
**Conditional mutual information with the root for noun (left) and verb (right) affix classes in optimized orderings across languages.** Affix classes that tend to be ordered closer to the root also tend to have higher mutual information with it.

The results are shown in Figure 10. In accordance with the principle of information locality, affixes that are ordered closer to the root in the two typological universals and in optimized orderings almost always have higher mutual information with the root than affixes ordered farther away from the root. For nouns, number has consistently higher mutual information with the root than case, that is, the identity of the root constrains number more strongly than case. Similarly, for verbs, the identity of the verb constrains the applicability of derivational affixes most, followed by valence and voice. Agreement affixes tend to have the lowest mutual information, that is, their identity is least constrained by the identity of the verb.

## DISCUSSION

We have examined morpheme ordering in nouns and verbs in six morphologically rich agglutinating languages, testing the recently proposed efficient tradeoff hypothesis (Hahn et al., 2021) as an explanatory account of morpheme ordering. We compared actual morpheme orderings to other possible orderings and to orderings optimized for efficiency of the memory-surprisal tradeoff. In most cases, we found that the real ordering provided more efficient tradeoffs than most alternative orderings. More importantly, we found that the real orderings match the optimal orderings with high accuracy, higher than the vast majority of other possible orderings. Beyond language-specific ordering patterns, optimization recovers previously documented language universals of morpheme order. These results support the idea that optimization for processing effort can explain universals of morpheme ordering, specifically Greenberg’s Universal 39 for nouns, and the ordering of valence, voice, TAM, and agreement affixes for verbs.

### Efficiency Optimization and Language Change

The efficient tradeoff hypothesis is compatible with different potential mechanisms through which languages come to exhibit efficient orderings (Hahn et al., 2021). One possibility is that speakers organize information in such a way as to facilitate comprehension (Brennan & Williams, 1995; Clark & Murphy, 1982; Lindblom, 1990). Another possibility is that efficient memory-surprisal tradeoffs result from the minimization of effort during sentence planning and production (Bock & Warren, 1985; Fedzechkina & Jaeger, 2020; Ferreira & Dell, 2000; MacDonald, 2013). While the memory-surprisal tradeoff is defined in terms of the comprehender’s memory load and processing difficulty, Hahn et al. (2021) prove an analogous result in sentence production. Efficient memory-surprisal tradeoffs might also facilitate language learning, if information locality makes it easier to learn generalizations from adjacent elements in language. Furthermore, the efficient tradeoff hypothesis may also be compatible with well-known grammaticalization processes, in particular, with processes of chunking and subsequent grammaticalization of frequent units (Bybee, 2006; Bybee & Hopper, 2001): when two items occur together very frequently, they will also tend to have high mutual information, and vice versa. Investigating in more detail how efficient orderings arise, possibly using diachronic data, is an interesting problem for future research.

### Limitations

Due to limitations in the availability of large-scale annotated text, this study builds on languages from Eurasia and Africa, not representing Australia and America. Among the languages, Hungarian and Finnish are genetically related, sharing a common ancestor about 5,000 years ago (Maurits et al., 2020). Some linguists also propose genetic or areal relations beyond these (particularly Japanese, Korean, Turkish), but any such genetic relations would have to be substantially more ancient. Importantly, the morphemes found in these languages as considered here are generally not cognate. Thus, the commonalities across languages found cannot be traced to inherited orderings of morphemes that are inherited from a common ancestor.

A limitation of this study is that memory-surprisal tradeoffs are estimated on finite datasets that do not cover all possible morphological forms of a language. However, to the extent that this limitation impacts the estimation of memory-surprisal tradeoffs, it should equally apply to real and counterfactual orderings. We thus do not expect the relative measured efficiencies of different orderings to be impacted by the finiteness of data.

### Relation to Previous Accounts

In this section, we relate our results to existing explanatory accounts of morpheme ordering reviewed in the section Previous Accounts of Morpheme Ordering. In a review of research on morpheme ordering, Manova and Aronoff (2010) categorize approaches to morpheme ordering into three classes (similarly Rice, 2000, 2011): orderings that are motivated by properties of syntax, semantics, or phonology (*grammatical theories*); orderings that are motivated by human language processing responding to statistical properties of language (*processing theories*); and orderings that are arbitrarily stipulated (*arbitrary orderings*). The efficient tradeoff hypothesis falls into the second class, explaining morpheme ordering based on minimization of human processing effort. In this section, we describe how it relates to other accounts across these three clusters, and show how the efficient tradeoff hypothesis has tight connections with notions proposed across seemingly very different accounts.

Bybee (1985) argues that morphemes that are semantically more relevant to the root are ordered closer to it. While semantic relevance and mutual information are a priori different notions, they may be related. For instance, according to Bybee, valence and voice markers are more relevant to the root than TAM markers, as they alter the verb’s argument structure (Bybee, 1985, p. 20). They have high mutual information with the verb stem (Figure 10), since only certain verbs (primarily transitive verbs) can form a passive voice. Indeed, Bybee (1985) suggests that highly relevant affixes tend to be less generally applicable, and vice versa.

A second prominent grammatical account is the scope-based account, which holds that morphemes are ordered in the order in which their meanings combine to form the meaning of the full word (Rice, 2000). In a study of noun phrase modifiers, Culbertson et al. (2020) argued that mutual information provides a statistical operationalization of scope in conceptual structure, that is, that elements have higher mutual information when they are closer together in conceptual structure. If this is true, then the efficient tradeoff hypothesis generally predicts ordering to respect scope relations.

However, it also predicts that orderings can deviate from conceptual structure depending on the statistics of the input. In particular, the efficient tradeoff hypothesis formally explains the finding that, in the artificial language learning experiments of Saldana et al. (2021), a preference for orderings following Greenberg’s Universal 39 could be reversed when the form of the case suffix depended on the noun: if the choice of case suffix depends on the noun, this increases the mutual information between the case suffix and the noun stem. As a consequence, orderings are more efficient when they place the case suffix closer to the noun stem.

Other grammatical accounts explain morpheme ordering in terms of a parallelism to word order, either through diachronic fossilization of words into affixes or through synchronic constraints on language (Baker, 1985; Givón, 1971; Vennemann, 1973). Unlike theories that only appeal to diachronic fossilization, the efficient tradeoff hypothesis accounts for observations that morpheme orderings respecting the universals do not always historically arise from previous ordering of words. As a theory of ordering at multiple levels, the efficient tradeoff hypothesis provides a cognitive motivation for proposed principles of parallelism between morpheme and word order (Baker, 1985), to the extent that the same statistical relations hold on the levels of morphemes and words.

The perhaps most prominent processing theory of morpheme ordering, the theory of complexity-based ordering (Hay, 2002; Hay & Baayen, 2005; Hay & Plag, 2004; Plag, 2002; Plag & Baayen, 2009), holds that affixes are closer to the root when they are less “separable,” where separability indicates the productivity of an affix and the likelihood that affixes are processed separately with the base in the dual processing race model of human lexical access, which asserts that morphological forms can be processed either separately in terms of its components or as a whole (Baayen, 1993). Unlike the other theories discussed here, this theory has primarily been applied to derivational suffixes in English, not to the crosslinguistic generalizations that we study here. Nonetheless, links can be established between this theory and the efficient tradeoff hypothesis. A prominent operationalization of the separability of an affix is in terms of relative frequencies: affixes are more likely to be processed together with the root when the composite form has a higher frequency compared to the base form (Hay, 2001). This has an interesting relation to mutual information: if the compound form is very frequent, in relation to the baseline frequencies of the base and the affix, then there is high (pointwise) mutual information between them. Conversely, if it is infrequent, mutual information is low. In this case, the prediction of the theory of complexity-based ordering is recovered by the efficient tradeoff hypothesis.

The efficient tradeoff hypothesis may also be related to the proposal of Inkelas (2016), who suggests that morphemes are closer to the root when they are less predictable from the preceding morpheme. The proposal received preliminary support in a pilot study of Turkish verbs. Depending on the details of usage statistics, this proposal and the efficient tradeoff hypothesis can be independent, contradictory, or even equivalent. For instance, if there is no systematic relationship between affixes’ mutual information with the root and with immediately surrounding affixes, the proposals might be independent. However, if affixes that have low mutual information with the root tend to have higher information with their immediately surrounding affixes, the two proposals can make similar or even equivalent predictions.

There are also studies suggesting that properties of morpheme ordering may be language-specific and essentially arbitrary. A classical approach to describing morpheme ordering is in terms of levels, where morphemes from a higher level occur before morphemes from a lower level (Siegel, 1979), and in terms of templates that describe the ordering of morphemes (Hyman, 2003; Inkelas, 1993; Nordlinger, 2010; Simpson & Withgott, 1986; Spencer, 1991; Stump, 1992). Ordering based on language-specific templates has been proposed specifically in cases where observed morpheme ordering is in conflict with semantic scope, as in Bantu languages (Hyman, 2003). Fabb (1988) prominently describes English affix ordering in terms of the selectional restrictions that individual affixes place on which other affixes they can attach to. While this approach does not make statements as to which affixes would go closer to the base in a given language, it does suggest that morpheme ordering is described based on the pairwise interactions between adjacent morphemes. In a similar vein, Ryan (2010) propose a model based on weighted bigram constraints in Tagalog, for the (rather uncommon) case of flexible morpheme ordering. Ordering constraints operating on adjacent pairs of morphemes might provide relatively efficient memory-surprisal tradeoffs because the appearance of a morpheme is constrained only by its immediately adjacent morphemes.

In conclusion, the efficient tradeoff hypothesis, while derived specifically in terms of processing effort, has close relations to various major theories of morpheme ordering, including those that make no reference to human processing. The efficient tradeoff hypothesis need not be seen as contradictory to any of those accounts. Rather, it may provide a unified account motivating each of those accounts: it motivates why constraints based on semantics (Bybee, 1985; Rice, 2000), word order (Baker, 1985; Givón, 1971; Vennemann, 1973), and usage frequencies (Hay, 2002; Inkelas, 2016; Plag, 2002; Plag & Baayen, 2009) all seem to impact morpheme ordering, from a single principle based on an information-theoretic analysis of incremental processing difficulty and memory load. It also makes predictions for ordering when those prior theories conflict, for instance, when semantic scope and locality of dependencies make opposing predictions (Saldana et al., 2021). In such cases, the relative strength with which those factors impact mutual information is predicted to determine which ordering is found. Beyond offering a unified account, it provides an explicit operationalization that is readily applied to new languages to make testable predictions, addressing a challenge faced by some of the previous accounts.

### Other Aspects of Morphology

We modeled morpheme ordering using slots representing grammatical categories. A more general approach could be based on individual morphemes, reflecting the fact that ordering is not always identical across all morphemes in a grammatical category (Mansfield et al., 2020); one example from the languages considered here is the third-person plural suffix in Turkish. Mansfield et al. (2020) argue for a typological universal called category clustering stating that markers of the same morphological categories tend to be expressed in the same position (see also Crysmann & Bonami, 2015; Stump, 2001). We expect that category clustering might produce more efficient memory-surprisal tradeoffs: morphemes that encode different values of the same grammatical feature are mutually exclusive, so that the appearance of one provides information about the (non-)appearance of the other. Future work could test the possibility that the graded nature of category clustering might in part arise from optimizing the efficiency of the memory-surprisal tradeoff.

Our study focused on agglutination, where a word carries multiple clearly separated morphemes with distinct functions. There are other types of morphological processes that deserve study. Many languages show fusion (Bickel & Nichols, 2013) where different categories are fused into a single morpheme, or stem changes, such as English *swim* → *swam*. An extreme case is nonconcatenative morphology (e.g., in Arabic, k-t-b “to write” forms *katab-* “wrote,” *-aktub* “write/be writing,” *-kutib-* “was written”). These types of morphological processes are not described in terms of the ordering of different morphemes. We leave it to future research to determine whether these processes are also constrained by cognitive considerations of processing efficiency.

While we have focused on the relative distance from the root, we have not touched on the question of why a morpheme is realized as a prefix or a suffix in a given language. There are well-known correlations between suffixing or prefixing preference and word order (Greenberg, 1963). It is an interesting problem for future research to study whether these correlations might arise from processing efficiency optimization, as has been proposed for the generally observed suffixing preference (Cutler et al., 1985; Himmelmann, 2014).

## CONCLUSION

We have tested the recently proposed efficient tradeoff hypothesis as a predictor of morpheme ordering with data from verbs and nouns across six languages. We found that attested morpheme orders provide more efficient tradeoffs than most other possible orderings and that many properties of observed orderings are recovered by optimizing for tradeoff efficiency. Across languages, we found that optimized orderings predict real orderings better than baselines, and in some languages almost perfectly. Optimization also successfully predicts prominent universals of morpheme ordering, both for nouns and verbs. These results support the efficient tradeoff hypothesis as a theory of order in language, and more broadly suggest that morpheme ordering reflects optimization of processing efficiency.

## FUNDING INFORMATION

National Science Foundation (https://dx.doi.org/10.13039/100000001), Award ID: 1950223.

## AUTHOR CONTRIBUTIONS

MH: Conceptualization: Lead; Formal analysis: Equal; Methodology: Lead; Visualization: Lead; Writing - Original Draft: Lead; Writing - Review & Editing: Equal. RM: Conceptualization: Supporting; Formal analysis: Equal; Visualization: Supporting; Writing - Original Draft: Supporting; Writing - Review & Editing: Equal. JD: Conceptualization: Supporting; Formal analysis: Supporting; Methodology: Supporting; Supervision: Lead; Visualization: Supporting; Writing - Original Draft: Supporting; Writing - Review & Editing: Equal.

## Note

^1^ In addition to the two universals discussed there, they also respect the universal that derivational affixes are closer to the stem than inflectional affixes mentioned in the Introduction.

## References

[bib1] Asahara, M., Kanayama, H., Tanaka, T., Miyao, Y., Uematsu, S., Mori, S., Matsumoto, Y., Omura, M., & Murawaki, Y. (2018). Universal dependencies version 2 for Japanese. In Proceedings of the Eleventh International Conference on Language Resources and Evaluation (LREC 2018). European Language Resources Association (ELRA).

[bib2] Aurnhammer, C., & Frank, S. L. (2019). Evaluating information-theoretic measures of word prediction in naturalistic sentence reading. Neuropsychologia, 134, Article 107198. 10.1016/j.neuropsychologia.2019.107198, 31553896

[bib3] Baayen, R. H. (1993). On frequency, transparency and productivity. In G. Booij & J. van Marle (Eds.), Yearbook of morphology 1992 (pp. 181–208). Springer. 10.1007/978-94-017-3710-4_7

[bib4] Baker, M. (1985). The mirror principle and morphosyntactic explanation. Linguistic Inquiry, 16(3), 373–416.

[bib5] Bauer, L. (2010). An overview of morphological universals. Word Structure, 3(2), 131–140. 10.3366/word.2010.0001

[bib6] Bickel, B., & Nichols, J. (2013). Fusion of selected inflectional formatives. In M. S. Dryer & M. Haspelmath (Eds.), The world atlas of language structures online. Max Planck Institute for Evolutionary Anthropology.

[bib7] Binnick, R. I. (1991). Time and the verb: A guide to tense and aspect. Oxford University Press.

[bib8] Binnick, R. I. (2012). The oxford handbook of tense and aspect. Oxford University Press. 10.1093/oxfordhb/9780195381979.001.0001

[bib9] Blevins, J. (2003). Passives and impersonals. Journal of Linguistics, 39, 473–520. 10.1017/S0022226703002081

[bib10] Bloomfield, L. (1926). A set of postulates for the science of language. Language, 2(3), 153–164. 10.2307/408741

[bib11] Bock, J. K., & Warren, R. K. (1985). Conceptual accessibility and syntactic structure in sentence formulation. Cognition, 21(1), 47–67. 10.1016/0010-0277(85)90023-X, 4075761

[bib12] Brennan, S. E., & Williams, M. (1995). The feeling of another’s knowing: Prosody and filled pauses as cues to listeners about the metacognitive states of speakers. Journal of Memory and Language, 34(3), 383–398. 10.1006/jmla.1995.1017

[bib13] Bybee, J. L. (1985). Morphology: A study of the relation between meaning and form. Benjamins. 10.1075/tsl.9

[bib14] Bybee, J. L. (2006). From usage to grammar: The mind’s response to repetition. Language, 82(4), 711–733. 10.1353/lan.2006.0186

[bib15] Bybee, J. L., & Hopper, P. J. (Eds.). (2001). Frequency and the emergence of linguistic structure. Benjamins. 10.1075/tsl.45

[bib16] Bybee, J. L., Perkins, R. D., & Pagliuca, W. (1994). The evolution of grammar: Tense, aspect, and modality in the languages of the world. University of Chicago Press.

[bib17] Caballero, G. (2010). Scope, phonology and morphology in an agglutinating language: Choguita rarámuri (tarahumara) variable suffix ordering. Morphology, 20(1), 165–204. 10.1007/s11525-010-9147-4

[bib18] Chun, J., Han, N., Hwang, J. D., & Choi, J. D. (2018). Building universal dependency treebanks in Korean. In N. Calzolari (Eds.), Proceedings of the Eleventh International Conference on Language Resources and Evaluation (LREC 2018), Miyazaki, Japan, May 7–12. European Language Resources Association (ELRA).

[bib19] Clark, H. H., & Murphy, G. L. (1982). Audience design in meaning and reference. In Advances in psychology (Vol. 9, pp. 287–299). Elsevier. 10.1016/S0166-4115(09)60059-5

[bib20] Cohen Priva, U. (2012). Sign and signal: Deriving linguistic generalizations from information utility (Doctoral dissertation). Stanford University.

[bib21] Coltekin, C., Cebiroglu Eryigit, G., Gokirmak, M., Kasikara, H., Sulubacak, U., & Tyers, F. (2020). UD Turkish-IMST, version 2.6. GitHub. https://github.com/UniversalDependencies/UD_Turkish-IMST

[bib22] Comrie, B. (1976). Aspect: An introduction to the study of verbal aspect and related problems. Cambridge University Press.

[bib23] Corbett, G. G. (2003). Agreement: Terms and boundaries. In Proceedings of the 2001 Texas Linguistic Society Conference, Austin, Texas (pp. 109–122). Texas Linguistic Forum.

[bib24] Cover, T. M., & Thomas, J. (2006). Elements of information theory. John Wiley & Sons. 10.1002/047174882X

[bib25] Crysmann, B., & Bonami, O. (2015). Variable morphotactics in information-based morphology. Journal of Linguistics, 52, 311–374. 10.1017/S0022226715000018

[bib26] Culbertson, J., Schouwstra, M., & Kirby, S. (2020). From the world to word order: Deriving biases in noun phrase order from statistical properties of the world. Language, 96(3), 696–717. 10.1353/lan.2020.0045

[bib27] Cutler, A., Hawkins, J., & Gilligan, G. (1985). The suffixing preference: A processing explanation. Linguistics, 23, 723–758. 10.1515/ling.1985.23.5.723

[bib28] Dahl, O. (1985). Tense and aspect systems. Oxford: Basil Blackwell.

[bib29] Davies, M. (2012). The corpus of contemporary American English (COCA). (https://www.english-corpora.org/coca/)

[bib30] de Courtenay, B. (1972). An attempt at a theory of phonetic alternations. In E. Stankiewicz (Ed.), A Baudouin de Courtenay anthology (pp. 144–212). Indiana University Press.

[bib31] Demuth, K. (1992). Acquisition of Sesotho. In D. I. Slobin (Ed.), The cross-linguistic study of language acquisition (pp. 557–638). Lawrence Erlbaum Associates.

[bib32] Doke, C. M., & Mofokeng, S. M. (1967). Textbook of southern Sotho grammar. Longmans.

[bib33] Dryer, M. S. (2013a). Coding of nominal plurality. In M. S. Dryer & M. Haspelmath (Eds.), The world atlas of language structures online. Max Planck Institute for Evolutionary Anthropology.

[bib34] Dryer, M. S. (2013b). Expression of pronominal subjects. In M. S. Dryer & M. Haspelmath (Eds.), The world atlas of language structures online. Max Planck Institute for Evolutionary Anthropology.

[bib35] Dryer, M. S. (2013c). Negative morphemes. In M. S. Dryer & M. Haspelmath (Eds.), The world atlas of language structures online. Max Planck Institute for Evolutionary Anthropology.

[bib38] Fabb, N. (1988). English suffixation is constrained only by selectional restrictions. Natural Language and Linguistic Theory, 6(4), 527–539. 10.1007/BF00134491

[bib39] Farkas, R., Simkó, K., Szántó, Z., Varga, V., & Vincze, V. (2020). UD Hungarian-Szeged, version 2.6. GitHub. https://github.com/UniversalDependencies/UD_Hungarian-Szeged

[bib40] Fedzechkina, M., & Jaeger, T. F. (2020). Production efficiency can cause grammatical change: Learners deviate from the input to better balance efficiency against robust message transmission. Cognition, 196, Article 104115. 10.1016/j.cognition.2019.104115, 31790998

[bib41] Ferreira, V. S., & Dell, G. S. (2000). Effect of ambiguity and lexical availability on syntactic and lexical production. Cognitive Psychology, 40(4), 296–340. 10.1006/cogp.1999.0730, 10888342

[bib42] Ferrer i Cancho, R. (2004). Euclidean distance between syntactically linked words. Physical Review E, 70(5), Article 056135. 10.1103/PhysRevE.70.056135, 15600720

[bib43] Frank, S., & Hoeks, J. (2019). The interaction between structure and meaning in sentence comprehension: Recurrent neural networks and reading times. In A. K. Goel, C. M. Seifert, & C. Freksa (Eds.), Proceedings of the 41th Annual Meeting of the Cognitive Science Society, COGSCI 2019: Creativity + Cognition + Computation, Montreal, Canada, July 24–27 (pp. 337–343). Cognitive Science Society.

[bib44] Frazier, L. (1985). Syntactic complexity. In D. R. Dowty, L. Karttunen, & A. Zwicky (Eds.), Natural language parsing: Psychological, computational, and theoretical perspectives (pp. 129–189). Cambridge University Press. 10.1017/CBO9780511597855.005

[bib45] Futrell, R., Gibson, E., & Levy, R. P. (2020). Lossy-context surprisal: An information-theoretic model of memory effects in sentence processing. Cognitive Science, 44(3), Article e12814. 10.1111/cogs.12814, 32100918 PMC7065005

[bib46] Futrell, R., Mahowald, K., & Gibson, E. (2015). Large-scale evidence of dependency length minimization in 37 languages. Proceedings of the National Academy of Sciences, 112(33), 10336–10341. 10.1073/pnas.1502134112, 26240370 PMC4547262

[bib47] Gibson, E. (1998). Linguistic complexity: Locality of syntactic dependencies. Cognition, 68(1), 1–76. 10.1016/S0010-0277(98)00034-1, 9775516

[bib49] Gildea, D., & Temperley, D. (2007). Optimizing grammars for minimum dependency length. In Proceedings of the 45th Annual Meeting of the Association of Computational Linguistics (pp. 184–191). Association for Computational Linguistics.

[bib50] Ginter, F., Kanerva, J., Laippala, V., Miekka, N., Missilä, A., Ojala, S., & Pyysalo, S. (2020). UD Finnish-TDT, version 2.6. GitHub. https://github.com/UniversalDependencies/UD_Finnish-TDT

[bib51] Givón, T. (1971). Historical syntax and synchronic morphology: An archaeologist’s field trip. CLS, 7, 349–415.

[bib52] Givón, T. (1985). Iconicity, isomorphism and non-arbitrary coding in syntax. In J. Haiman (Ed.), Iconicity in syntax (pp. 187–219). Benjamins. 10.1075/tsl.6.10giv

[bib53] Goodkind, A., & Bicknell, K. (2018). Predictive power of word surprisal for reading times is a linear function of language model quality. In A. B. Sayeed, C. Jacobs, T. Linzen, & M. V. Schijndel (Eds.), Proceedings of the 8th Workshop on Cognitive Modeling and Computational Linguistics (CMCL 2018), Salt Lake City, Utah, January 7, 2018 (pp. 10–18). Association for Computational Linguistics. 10.18653/v1/W18-0102

[bib54] Greenberg, J. H. (1963). Some universals of grammar with particular reference to the order of meaningful elements. In J. H. Greenberg (Ed.), Universals of language (pp. 73–113). MIT Press.

[bib55] Hahn, M., Degen, J., & Futrell, R. (2021). Modeling word and morpheme order in natural language as an efficient tradeoff of memory and surprisal. Psychological Review, 128(4), 726–756. 10.1037/rev0000269, 33793259

[bib56] Hale, J. T. (2001). A probabilistic Earley parser as a psycholinguistic model. In Proceedings of the Second Meeting of the North American Chapter of the Association for Computational Linguistics and Language Technologies (pp. 1–8). The Association for Computational Linguistics. 10.3115/1073336.1073357

[bib57] Hammarström, H., Forkel, R., Haspelmath, M., & Bank, S. (2021). Glottolog 4.4. Max Planck Institute for Evolutionary Anthropology. 10.5281/zenodo.4761960

[bib58] Hasegawa, Y. (2014). Japanese: A linguistic introduction. Cambridge University Press. 10.1017/CBO9781139507127

[bib59] Haspelmath, M. (1993). The diachronic externalization of inflection. Linguistics, 31(2), 279–310. 10.1515/ling.1993.31.2.279

[bib60] Hawkins, J. A. (1994). A performance theory of order and constituency. Cambridge University Press. 10.1017/CBO9780511554285

[bib61] Hawkins, J. A. (2003). Efficiency and complexity in grammars: Three general principles. In J. Moore & M. Polinsky (Eds.), The nature of explanation in linguistic theory (pp. 121–152). CSLI Publications.

[bib62] Hay, J. (2001). Lexical frequency in morphology: Is everything relative? Linguistics, 39(6), 1041–1070. 10.1515/ling.2001.041

[bib63] Hay, J. (2002). From speech perception to morphology: Affix ordering revisited. Language, 78(3), 527–555. 10.1353/lan.2002.0159

[bib64] Hay, J., & Baayen, H. (2005). Shifting paradigms: Gradient structure in morphology. Trends in Cognitive Sciences, 9(7), 342–348. 10.1016/j.tics.2005.04.002, 15993361

[bib65] Hay, J., & Plag, I. (2004). What constrains possible suffix combinations? On the interaction of grammatical and processing restrictions in derivational morphology. Natural Language and Linguistic Theory, 22(3), 565–596. 10.1023/B:NALA.0000027679.63308.89

[bib66] Himmelmann, N. (2014). Asymmetries in the prosodic phrasing of function words: Another look at the suffixing preference. Language, 90, 927–960. 10.1353/lan.2014.0105

[bib67] Huddleston, R., & Pullum, G. (2002). The Cambridge grammar of the English language. Cambridge University Press. 10.1017/9781316423530

[bib68] Hyman, L. M. (2003). Suffix ordering in Bantu: A morphocentric approach. In G. Booij & J. van Marle (Eds.), Yearbook of morphology 2002 (pp. 245–281). Springer. 10.1007/0-306-48223-1_8

[bib69] Inkelas, S. (1993). Nimboran position class morphology. Natural Language and Linguistic Theory, 11(4), 559–624. 10.1007/BF00993014

[bib70] Inkelas, S. (2016). Affix ordering in optimal construction morphology. In D. Siddiqi & H. Harley (Eds.), Morphological metatheory (pp. 479–512). Benjamins. 10.1075/la.229.16ink

[bib71] Just, M. A., & Carpenter, P. A. (1992). A capacity theory of comprehension: Individual differences in working memory. Psychological Review, 99(1), 122–149. 10.1037/0033-295X.99.1.122, 1546114

[bib72] Karlsson, F. (1999). Finnish: An essential grammar. Routledge.

[bib73] Katamba, F. (2006). Morphology. Macmillan. 10.1007/978-1-137-11131-9

[bib74] Korotkova, N., & Lander, Y. (2010). Deriving affix ordering in polysynthesis: Evidence from adyghe. Morphology, 20(2), 299–319. 10.1007/s11525-010-9185-y

[bib75] Kuperberg, G. R., & Jaeger, T. F. (2016). What do we mean by prediction in language comprehension? Language, Cognition and Neuroscience, 31(1), 32–59. 10.1080/23273798.2015.1102299, 27135040 PMC4850025

[bib76] Levy, R. (2008). Expectation-based syntactic comprehension. Cognition, 106(3), 1126–1177. 10.1016/j.cognition.2007.05.006, 17662975

[bib77] Lewis, R. L., & Vasishth, S. (2005). An activation-based model of sentence processing as skilled memory retrieval. Cognitive Science, 29(3), 375–419. 10.1207/s15516709cog0000_25, 21702779

[bib78] Lindblom, B. (1990). Explaining phonetic variation: A sketch of the H&H theory. In Speech production and speech modelling (pp. 403–439). Springer. 10.1007/978-94-009-2037-8_16

[bib79] Liu, H. (2008). Dependency distance as a metric of language comprehension difficulty. Journal of Cognitive Science, 9(2), 159–191. 10.17791/jcs.2008.9.2.159

[bib80] Liu, H., Xu, C., & Liang, J. (2017). Dependency distance: A new perspective on syntactic patterns in natural languages. Physics of Life Reviews, 21, 171–193. 10.1016/j.plrev.2017.03.002, 28624589

[bib81] MacDonald, M. C. (2013). How language production shapes language form and comprehension. Frontiers in Psychology, 4, Article 226. 10.3389/fpsyg.2013.00226, 23637689 PMC3636467

[bib82] MacDonald, M. C., & Christiansen, M. H. (2002). Reassessing working memory: Comment on Just and Carpenter (1992) and Waters and Caplan (1996). Psychological Review, 109(1), 35–54. 10.1037/0033-295X.109.1.35, 11863041

[bib83] Manova, S., & Aronoff, M. (2010). Modeling affix order. Morphology, 20(1), 109–131. 10.1007/s11525-010-9153-6

[bib84] Mansfield, J., Stoll, S., & Bickel, B. (2020). Category clustering: A probabilistic bias in the morphology of verbal agreement marking. Language, 96, 255–293. 10.1353/lan.2020.0021

[bib85] Marr, D. (1982). Vision: A computational investigation into the human representation. W. H. Freeman.

[bib86] Maurits, L., de Heer, M., Honkola, T., Dunn, M., & Vesakoski, O. (2020). Best practices in justifying calibrations for dating language families. Journal of Language Evolution, 5(1), 17–38. 10.1093/jole/lzz009

[bib87] McElree, B., Foraker, S., & Dyer, L. (2003). Memory structures that subserve sentence comprehension. Journal of Memory and Language, 48(1), 67–91. 10.1016/S0749-596X(02)00515-6

[bib88] Mithun, M. (1994). Affixation and morphological longevity. In G. Booij & J. van Marle (Eds.), Yearbook of morphology (pp. 73–97). Springer. 10.1007/978-94-017-3714-2_3

[bib89] Mithun, M. (2000). The reordering of morphemes. In S. Gildea (Ed.), Reconstructing grammar: Comparative linguistics and grammaticalization (p. 231). Benjamins. 10.1075/tsl.43.09mit

[bib90] Narrog, H. (2010). The order of meaningful elements in the japanese verbal complex. Morphology, 20(1), 205–237. 10.1007/s11525-010-9150-9

[bib91] Nivre, J., de Marneffe, M., Ginter, F., Hajic, J., Manning, C. D., Pyysalo, S., Schuster, S., Tyers, F. M., & Zeman, D. (2020). Universal dependencies v2: An evergrowing multilingual treebank collection. In N. Calzolari (Eds.), Proceedings of the 12th Language Resources and Evaluation Conference (LREC 2020), Marseille, France, May 11–16 (pp. 4034–4043). European Language Resources Association.

[bib92] Nordlinger, R. (2010). Verbal morphology in murrinh-patha: Evidence for templates. Morphology, 20(2), 321–341. 10.1007/s11525-010-9184-z

[bib93] Palmer, F. R. (2001). Mood and modality (2nd ed.). Cambridge University Press. 10.1017/CBO9781139167178

[bib94] Piitulainen, J., & Nurmi, H. (2020). UD finnish-ftb, version 2.6. GitHub. https://github.com/UniversalDependencies/UD_Finnish-FTB

[bib95] Plag, I. (2002). The role of selectional restrictions, phonotactics and parsing in constraining suffix ordering in English. In G. Booij & J. van Marle (Eds.), Yearbook of morphology 2001 (pp. 285–314). Springer. 10.1007/978-94-017-3726-5_11

[bib96] Plag, I., & Baayen, H. (2009). Suffix ordering and morphological processing. Language, 85(1), 109–152. 10.1353/lan.0.0087

[bib97] Portner, P. (2018). Mood. Oxford University Press. 10.1093/oso/9780199547524.001.0001

[bib98] Rice, K. (2000). Morpheme order and semantic scope: Word formation in the Athapaskan verb. Cambridge University Press. 10.1017/CBO9780511663659

[bib99] Rice, K. (2011). Principles of affix ordering: An overview. Word Structure, 4(2), 169–200. 10.3366/word.2011.0009

[bib100] Rijkhoff, J. (1986). Word order universals revisited: The principle of head proximity. Belgian Journal of Linguistics, 1, 95–125. 10.1075/bjl.1.05rij

[bib101] Rounds, C. H. (2001). Hungarian: An essential grammar. Routledge.

[bib102] Ryan, K. M. (2010). Variable affix order: Grammar and learning. Language, 86(4), 758–791. 10.1353/lan.2010.0032

[bib103] Saldana, C., Oseki, Y., & Culbertson, J. (2021). Cross-linguistic patterns of morpheme order reflect cognitive biases: An experimental study of case and number morphology. Journal of Memory and Language, 118, Article 104204. 10.1016/j.jml.2020.104204

[bib104] Shore, S. (1988). On the so-called finnish passive. WORD, 39, 151–176. 10.1080/00437956.1988.11435787

[bib105] Siegel, D. C. (1979). Topics in English morphology. Massachusetts Institute of Technology.

[bib106] Simpson, J., & Withgott, M. (1986). Pronominal clitic clusters and templates. In H. Borer (Ed.), The syntax of pronominal clitics (pp. 147–174). Brill. 10.1163/9789004373150_008

[bib107] Smith, N. J., & Levy, R. (2013). The effect of word predictability on reading time is logarithmic. Cognition, 128(3), 302–319. 10.1016/j.cognition.2013.02.013, 23747651 PMC3709001

[bib108] Song, J. J. (2013). Nonperiphrastic causative constructions. In M. S. Dryer & M. Haspelmath (Eds.), The world atlas of language structures online. Max Planck Institute for Evolutionary Anthropology.

[bib109] Spencer, A. (1991). Morphological theory: An introduction to word structure in generative grammar. Blackwell.

[bib110] Spencer, A. (2006). Morphological universals. In R. Mairal & J. Gil (Eds.), Linguistic universals. Cambridge University Press. 10.1017/CBO9780511618215.006

[bib111] Stump, G. (2001). Inflectional morphology: A theory of paradigm structure. Cambridge University Press. 10.1017/CBO9780511486333

[bib112] Stump, G. T. (1992). On the theoretical status of position class restrictions on inflectional affixes. In G. Booij & J. van Marle (Eds.), Yearbook of morphology 1991 (pp. 211–241). Springer. 10.1007/978-94-011-2516-1_13

[bib113] Temperley, D., & Gildea, D. (2018). Minimizing syntactic dependency lengths: Typological/cognitive universal? Annual Review of Linguistics, 4, 1–15. 10.1146/annurev-linguistics-011817-045617

[bib114] van Schaaik, G. (2020). The Oxford Turkish grammar. Oxford University Press. 10.1093/oso/9780198851509.001.0001

[bib115] Vennemann, T. (1973). Explanation in syntax. In J. Kimball (Ed.), Syntax and semantics 2 (pp. 1–50). Seminar Press. 10.1163/9789004368804_002

[bib116] Wilcox, E. G., Gauthier, J., Hu, J., Qian, P., & Levy, R. P. (2020). On the predictive power of neural language models for human real-time comprehension behavior. In S. Denison, M. Mack, Y. Xu, & B. C. Armstrong (Eds.), Proceedings of the 42nd Annual Conference of the Cognitive Science Society (pp. 1707–1713). Cognitive Science Society.

[bib117] Yeon, J., & Brown, L. (2010). Korean: A comprehensive grammar. Routledge.

