# Supplementary material for: Morpheme Ordering Across Languages Reflects Optimization for Processing Efficiency
Source: Open Mind (Camb). 2022 Feb 9;5:208–32. doi: 10.1162/opmi_a_00051 (PMC9692062; doi:10.1162/opmi_a_00051)
Supplement: Supplementary file 1 [file opmi-05-208-s001.pdf]

# Supplementary Information: Morpheme Ordering across Languages Reflects Optimization for Processing Efficiency

Michael Hahn, Rebecca Mathew, Judith Degen

November 18, 2021

## Contents


**S1 Identifying Morphemes from Corpus Data** **1**
- S1.1 Korean Verb Suffixes . . . . . 1
- S1.2 Finnish Verb Inflection . . . . . 7
- S1.3 Turkish Verb Suffixes . . . . . 7
- S1.4 Hungarian Verb Suffixes . . . . . 7
- S1.5 Sesotho . . . . . 10
- S1.6 Japanese . . . . . 10
- S1.7 Nouns in Finnish. Hungarian, and Turkish . . . . . 12

**S2 Other Measures of Accuracy** **12**

**S3 Technical Details** **13**
- S3.1 Computation of Memory-Surprisal Tradeoff . . . . . 13
- S3.2 Example Computation of Memory-Surprisal Tradeoff . . . . . 16


## S1 Identifying Morphemes from Corpus Data

Here, we describe how we identified morphemes from corpus data and arrived at the classification of morphemes in the six languages as described in the main paper.

### S1.1 Korean Verb Suffixes

Korean verb morphology is very complex, and there is no generally agreed-upon description in terms of morphemes and slots. We extracted suffixes based on the annotation found in the corpus we used (Kaist corpus, Chun et al. [2018]) and the linguistic literature on Korean [Yeon and Brown, 2010]. The Kaist corpus already provides segmentation into morphemes; we postprocessed it in two ways: First, we made it more fine-grained by splitting morphemes that are merged in the annotation, and, second, we abstracted away consistently from allomorphy. For instance, the Kaist corpus separately labels the segments ㅂ니다 *-mnida* (as in 합니다 *hamnida* 'does') and 습니다 *-seumnida* (as in 했습니다 *hatseumnida* 'did'). These two segments actually are allomorphs, conditioned by the preceding material. Furthermore, they can both be segmented into the formal marker ㅂ/습 (*-p/-seup*, we label this underlying morpheme "$\mathrm{P}_5$", see below for

this notation), and the mood markers 니 (*-ni*, our NI$_6$) and 다 (*-da*, our DA$_7$). We therefore transform both segments into the abstract morpheme sequence P$_5$-NI$_6$-DA$_7$ (see below for this notation). We then partitioned the resulting morphemes into slots that make it possible to consistently describe the ordering of all forms encountered in the corpus. We indexed morphemes by a small-caps representation of a stylized phonological representation (such as P for -ㅂ/습 *-p/seup*),[1] with a subscript indicating their slot (such as P$_5$-NI$_6$-DA$_7$). With this procedure, we identified the slots described below:

1. Root
2. Derivation: The two derivational suffixes are *ha* ([Yeon and Brown, 2010, 4.1.2]) and the predicative *i*, whose function is similar to that of a copula [Yeon and Brown, 2010, 4.1.4].
3. Honorific SI$_3$ [Yeon and Brown, 2010, 4.3.2, 4.4.1]
4. Tense/Aspect suffixes include -ESS$_4$ for past [Yeon and Brown, 2010, 4.5.1.1], -ESSESS$_4$ for remote past [Yeon and Brown, 2010, 4.5.1.2], -GET$_4$ for future [Yeon and Brown, 2010, 4.5.2.1]
5. Formality P$_5$ (allomorphs include *-p-*, *-m-*, *-seum-*, [Yeon and Brown, 2010, 4.3.2])
6. Mood 1: We partition Mood suffixes into two slots, as these can be combined into strings of combined mood suffixes. Frequent elements of the Mood I slot are -N$_6$-, -NI$_6$- [Yeon and Brown, 2010, 4.3.2].
7. Mood II: Frequent elements of the Mood II slot are declarative -DA$_7$- [Yeon and Brown, 2010, 4.3.2], command -RA-, interrogative -KA-, the suffix -JI [Yeon and Brown, 2010, 4.2.2-3], and informal -EO$_7$-.
8. Connective endings, such as -GE$_8$, --SE$_8$, and many others
9. Polite -YO$_9$
10. Nominalizers, such as -GI$_{10}$ [Yeon and Brown, 2010, 2.2].

We show morphemes occurring at least 50 times in slots 2-8 in Figure S1. We do not provide the list of the (very numerous) conjunctive and nominalizing suffixes as these are not the focus of the typological generalization that we are interested in. Additional morphemes that do not fit into any of the slots and occur less than 50 times are placed into an UNKNOWN slot.

Next, we illustrate how our segmentation corresponds to commonly described paradigms. First, in Table S2, we describe the correspondence to the speech style system described by [Yeon and Brown, 2010, 4.3.2]. Second, in Tables S3–S5, we show an illustrative paradigm for the verb 하다 *hada* 'to do' as described in Wiktionary[2], which covers some of the affixes in slots 4–7. In Table S6, we show the part of the paradigm of the verb *ida* 'to be' corresponding to Table S3 (the other parts of the paradigm are analogous to those of *hada*). Note that none of these paradigms constitute exhaustive lists of all forms of these verbs; instead, they represent commonly used forms in a systematic paradigmatic representation. We show these in order to illustrate how commonly used forms map onto morpheme sequences.

[1]These transcriptions are purely conventional, we do not intend these to correspond to a theory of how underlying phonological forms are realized as surface phone strings.

[2]Selection of forms and labeling of rows and columns in Tables S3–S6 are from `https://en.wiktionary.org/wiki/하다` and `https://en.wiktionary.org/wiki/있다` (retrieved September 16, 2020).

| Slot | Morphs | Short | Description | Citation |
|---|---|---|---|---|
| Derivation | 하 | $\text{HA}_2$ | | [Yeon and Brown, 2010, 4.1.2] |
| | 이 | $\text{I}_2$ | | [Yeon and Brown, 2010, 4.1.4] |
| Honorific | 시 | $\text{SI}_3$ | | [Yeon and Brown, 2010, 4.3.2, 4.4.1] |
| Tense/Asp. | 었었 | $\text{ESSESS}_4$ | | [Yeon and Brown, 2010, 4.5.1.2] |
| | 겠 | $\text{GET}_4$ | assertive | [Yeon and Brown, 2010, 4.5.2.1] |
| | ㅆ | $\text{ESS}_4$ | past | [Yeon and Brown, 2010, 4.5.1.1] |
| Formality | ㅂ | $\text{P}_5$ | formal-polite | [Yeon and Brown, 2010, 4.3.2] |
| Mood 1 | 리 | $\text{RI}_6$ | | |
| | 니 | $\text{NI}_6$ | See Table S3 | [Yeon and Brown, 2010, 4.3.2] |
| | ㄴ | $\text{N}_6$ | See Table S3 | |
| Mood 2 | 시다 | $\text{SIDA}_7$ | Hortative, formal, polite | |
| | 어 | $\text{EO}_7$ | Indicative, informal | |
| | 자 | $\text{JA}_7$ | Hortative | [Yeon and Brown, 2010, 4.3.6.3] |
| | 소 | $\text{SO}_7$ | See Table S2 | |
| | ㄹ까 | $\text{LKKA}_7$ | Interrogative | [Yeon and Brown, 2010, 8.9] |
| | 오 | $\text{O}_7$ | See Table S2 | |
| | 지 | $\text{JI}_7$ | | [Yeon and Brown, 2010, 4.2.2-3] |
| | 까 | $\text{KA}_7$ | Interrogative | [Yeon and Brown, 2010, 4.3.4, p. 175; p. 183] |
| | 라 | $\text{RA}_7$ | command | [Yeon and Brown, 2010, 4.3.6.4] |
| | 다 | $\text{DA}_7$ | Declarative | [Yeon and Brown, 2010, 4.3.2] |
| Polite | 요 | $\text{YO}_9$ | See Table S2 | |

Table S1: Frequent Korean verb suffixes in slots 1-8. The first column describes the nine slots. Second column: Representation of one allomorph in Hangul. Third column: Identifier.

| | Statement | Question | Command | Proposal |
|---|---|---|---|---|
| Formal | ㅂ니다<br>-mnida<br>-P5-NI6-DA7 | ㅂ니까<br>-mnikka<br>-P5-NI6-KKA7 | 지-ㅂ-지오<br>-sipsio<br>-SI3-P5-SIO7 | 지-ㅂ-지다<br>-sipsida<br>-SI3-P5-SIDA7 |
| Polite | 아/어/요<br>-a/eoyo<br>-EO7-YO9 | | | |
| Semi-Formal | 오/소<br>-o/so<br>-O7/-SO7 | | 오<br>-o<br>-O7 | -ㅂ-지다<br>-p-sida<br>-P5-SIDA7 |
| Familiar | 네<br>-ne<br>-NE7 | 나/는가<br>-na/neunka<br>-NA7/-NEUNKA7 | 게<br>-ge<br>-GE8 | 세<br>-se<br>-SE8 |
| Intimate | 아/어<br>-a/eo<br>-EO7 | | | |
| Plain | 다<br>-da<br>-DA7 | (느)냐<br>-(neu)nya<br>-NYA7 | 라<br>-ra<br>-RA7 | 자<br>-ja<br>-JA7 |

Table S2: Correspondence between our morpheme segmentation and the speech style system described by [Yeon and Brown, 2010, 4.3.2]. In each cell, we provide the Hangul ending given by [Yeon and Brown, 2010], a transliteration, and a representation in terms of underlying morphemes.

| | | Formal non-polite | Informal non-polite | Informal polite | Formal polite |
|---|---|---|---|---|---|
| Indicative | Non-past | 한다<br>handa<br>-N6-DA7<br>하+ㄴ다<br>px+ef | 해<br>hae<br>-EO7<br>하+어<br>pvg+ecs | 해요<br>haeyo<br>-EO7-YO9<br>하+어+요<br>pvg+ef+jxf | 합니다<br>hamnida<br>-P5-NI6-DA7<br>하+ㅂ니다<br>pvg+ef |
| | Past | 했다<br>haet-da<br>-ESS4-DA7<br>하+었+다<br>ncpa+xsv+ep+ef | 했어<br>haesseo<br>-ESS4-EO7<br>하+었+어<br>px+ep+ef | 했어요<br>haesseoyo<br>-ESS4-EO7-YO9<br>하+었+어+요<br>px+ep+ef+jxf | 했습니다<br>haetseumnida<br>-ESS4-P5-NI6-DA7<br>하+었+습니다<br>pvg+ep+ef |
| Interrogative | Non-past | 하느냐<br>haneunya<br>-NYA7<br>하+느냐<br>px+ef | 해<br>hae<br>-EO7 | 해요<br>haeyo<br>-EO7-YO9 | 합니까<br>hamnikka<br>-P5-NI6+KKA7<br>하+ㅂ니까<br>px+ef |
| | Past | 했느냐<br>ha-n-neunya<br>-ESS4-NYA7<br>하+었+느냐<br>+ep+ef | 했어<br>hae-sseo<br>-ESS4-EO7<br>하+었+어<br>+ep+ef | 했어요<br>hae-sseo-yo<br>-ESS4-EO7-YO9<br>하+었+어+요<br>px+ep+ef+jxf | 했습니까<br>haetseumnikka<br>-ESS4-P5-NI6-KKA7<br>하+었+습니까<br>pvg+ep+ef |
| Hortative | | 하자<br>haja<br>-JA7<br>+자<br>+ef | 해<br>hae<br>-EO7 | 해요<br>haeyo<br>-EO7-YO9<br>하+어+요<br>pvg+ef+jxf | 합시다<br>hapsida<br>-P5-SIDA7<br>하+ㅂ시다<br>ef |
| Imperative | | 해라, 하여라<br>haera, hayeora<br>-RA7<br>하+어라, 하+어라<br>pvg+ef, pvg+ef | 해<br>hae<br>-EO7 | 해요<br>haeyo<br>-EO7-YO9 | 합시오<br>hapsio<br>-P5-SI3O<br>+ㅂ시오<br>+ef |
| Assertive | | 하겠다<br>hagetda<br>-GET4-DA7<br>하+겠+다<br>px+ep+ef | 하겠어<br>hagesseo<br>-GET4-EO7<br>하+겠+어<br>pvg+ep+ef | 하겠어요<br>hagesseoyo<br>-GET4-EO7-YO9<br>하+겠+어+요<br>pvg+ep+ef+jxf | 하겠습니다<br>hagetseumnida<br>GET4-P5-NI6-DA7<br>하+겠+습니다<br>pvg+ep+ef |

Table S3: Selected forms of *hada* 'to do' (see text for further information). In each cell, we provide Hangul and transliterated (e.g., handa) forms, a representation in terms of underlying morphemes (e.g., -N6-DA7), and – where available – how the form is segmented in the Kaist corpus (e.g., px+ef).

| | Formal non-polite | Informal non-polite | Informal polite | Formal polite |
|---|---|---|---|---|
| Cause/Reason | 해<br>hae<br>-EO7 | 해서, 하여서<br>haeseo, hayeoseo<br>-EOSEO8<br>하+어서, 하+어서<br>pvg+ecs, xsm+ecs | 하니<br>hani<br>-NI8<br>하+니<br>pvg+ecs | 하니까<br>hanikka<br>-NIKKA8<br>하+니까<br>xsm+ef |
| Contrast | 하지만<br>hajiman<br>-JIMAN8 | 하는데<br>haneunde<br>-NEUNDE8 | 하더니<br>haedoni<br>-DEONI8 | |
| Conjunction | 하고<br>hago<br>-GO8 | | | |
| Condition | 하면<br>hamyeon<br>-MYEON8 | 해야, 하여야<br>haeya, hayeoya<br>-EOYA8 | | |
| Motive | 하려고<br>haryeogo<br>-RYEOGO8 | | | |

Table S4: Continuation of Table S3: Forms of *hada* with a conjunctive ending.

| | | Formal non-polite | Informal non-polite | Informal polite | Formal polite |
|---|---|---|---|---|---|
| Indicative | Non-past | 하신다<br>-SI3-N6-DA7<br>하+시+ㄴ다 | 하셔<br>SI3-EO7<br>– | 하세요, 하셔요<br>SI3-EO7-YO<br>하+시+어+요 | 하십니다<br>SI3-P5-NI6-DA7<br>하+시+ㅂ니다 |
| | | 하셨다<br>SI3-ESS4-DA7<br>하+셨+다 | 하셨어<br>SI3-ESS4-EO7<br>– | 하셨어요<br>SI3-ESS4-EO7-YO<br>– | 하셨습니다<br>SI3-ESS4-P5-NI6-DA7<br>하+셨+습니다 |
| Interrogative | Non-past | 하시느냐<br>SI3-NYA7<br>– | 하셔<br>SI3-EO7<br>– | 하세요, 하셔요<br>SI3-EO7-YO<br>하+시+어+요 | 하십니까<br>SI3-P5-NI6-KKA7<br>– |
| | Past | 하셨느냐<br>SI3-ESS4-NYA7 | 하셨어<br>SI3-ESS4-EO7 | 하셨어요<br>SI3-ESS4-EO7-YO | 하셨습니까<br>SI3-ESS4-P5-NI6-KKA7 |
| Imperative | | 하시라<br>-SI3-RA | 하셔<br>-SI3-EO7<br>하+시+어+ | 하세요<br>-SI3-EO7-YO<br>하+십시오 | 하십시오<br>-SI3-P5-SI3O |
| Assertive | | 하시겠다<br>-SI3-GET4-DA7<br>– | 하시겠어<br>-SI3-GET4-EO7<br>– | 하시겠어요<br>-SI3-GET4-EO7-YO<br>– | 하시겠습니다<br>-SI3-GET4-P5-NI6-DA7<br>– |

Table S5: Continuation of Tables S3–S4: Selected forms of hada with an honorific (SI3). We provide Hangul forms, morpheme sequences, and – where available – segmentations as given in the Kaist corpus.

| | | Formal non-polite | Informal non-polite | Informal polite | Formal polite |
|---|---|---|---|---|---|
| Indicative | Non-past | 있다<br>있+다<br>-DA7<br>+ef | 있어<br>있+어<br>-EO7<br>+ef | 있어요<br>있+어+요<br>-EO7-YO<br>+ef+jxf | 있습니다<br>있+습니다<br>-P5-NI6-DA7<br>+ef |
| | Past | 있었다<br>있+었+다<br>-ESS4-DA7<br>+ep+ef | 있었어<br>있+었+어<br>ESS4-EO7<br>+ep+ef | 있었어요<br>있+었+어+요<br>ESS4-EO7-YO<br>+ep+ef+jxf | 있었습니다<br>있+었+습니다<br>-ESS4-P5-NI6-DA7<br>+ep+ef |
| Interrogative | Non-past | 있느냐<br>-NYA7<br>있+느냐<br>+ef | 있어<br>-EO7<br>있+어<br>+ef | 있어요<br>-EO7-YO<br>있+어+요<br>+ef+jxf | 있습니까<br>-P5-NI6-KKA7<br>있+습니까<br>+ef |
| | Past | 있었느냐<br>-ESS4-NYA7<br>있+었+느냐<br>+ep+ef | 있었어<br>-ESS4-EO7<br>있+었+어<br>paa+ep+ef | 있었어요<br>-ESS4-EO7-YO<br>있+었+어+요<br>paa+ep+ef+jxf | 있었습니까<br>-ESS4-P5-NI6-KKA7 |
| Assertive | | 있겠다<br>-GET4-DA7<br>있+겠+다<br>+ep+f | 있겠어<br>-GET4-EO7 | 있겠어요<br>-GET4-EO7-YO<br>있+겠+어+요<br>paa+ep+ef+jxf | 있겠습니다<br>-GET4-P5-NI6-DA7<br>있+겠+습니다<br>paa+ep+ef |

Table S6: Selected forms of *itda*. Refer to Table S3 for details.

## S1.2 Finnish Verb Inflection

In the Finnish corpus, verb forms are annotated using collections of features, such as `Mood=Ind` and `Number=Sing`, which make it pssible to uniquely map the form to a cell of the paradigm in Table S7.[3]. Additionally, Finnish verbs can take various final clitics [Karlsson, 1999, Section 91].

## S1.3 Turkish Verb Suffixes

In Table S8, we show the list of identified Turkish verb suffixes [van Schaaik, 2020].[4] In Figure S9, we illustrate this using selected forms of *yapmak*, covering TAM and agreement suffixes.[5]

## S1.4 Hungarian Verb Suffixes

In Table S10, we show the list of identified Hungarian verb suffixes [Rounds, 2001]. In Figure S11, we illustrate these using selected forms of three verbs.[6]

[3]For illustration, we provide forms of *tulla* from `https://en.wiktionary.org/wiki/tulla#Conjugation`, retrieved January 10, 2021.

[4]Note that there are some further derivational forms, such as reciprocal *-(I)ş* as in görüş- 'to meet' derived from gör- 'to see'. However, these are not annotated in the Universal Dependencies data, which treats the derived form as the lemma.

[5]The selection of forms follows: `https://en.wiktionary.org/wiki/yapmak#Turkish`, retrieved September 5, 2020.

[6]The selection of forms follows `https://en.wiktionary.org/wiki/tesz`, `https://en.wiktionary.org/wiki/csinál`, `https://en.wiktionary.org/wiki/ismer`, retrieved September 5, 2020.

| TAM | Agreement | Form | Suffixes |
|---|---|---|---|
| present tense | 1st sing | tulen | $\text{-n}_{\text{AGR}}$ |
| [Karlsson, 1999, Section 24] | 2nd sing. | tulet | $\text{-t}_{\text{AGR}}$ |
| | 3rd sing. | tulee | $\text{-V}_{\text{AGR}}$ |
| | 1st plur. | tulemme | $\text{-mme}_{\text{AGR}}$ |
| | 2nd plur. | tulette | $\text{-tte}_{\text{AGR}}$ |
| | 3rd plur. | tulevat | $\text{-vat}_{\text{AGR}}$ |
| | passive | tullaan | $\text{-t}_{\text{PASSIVE}}\text{-Vn}_{\text{AGR}}$ |
| past tense | 1st sing. | tulin | $\text{-i}_{\text{TAM}}\text{-n}_{\text{AGR}}$ |
| [Karlsson, 1999, Section 60] | 2nd sing. | tulit | $\text{-i}_{\text{TAM}}\text{-t}_{\text{AGR}}$ |
| | 3rd sing. | tuli | $\text{-i}_{\text{TAM}}\text{-V}_{\text{AGR}}$ |
| | 1st plur. | tulimme | $\text{-i}_{\text{TAM}}\text{-mme}_{\text{AGR}}$ |
| | 2nd plur. | tulitte | $\text{-i}_{\text{TAM}}\text{-tte}_{\text{AGR}}$ |
| | 3rd plur. | tulivat | $\text{-i}_{\text{TAM}}\text{-vat}_{\text{AGR}}$ |
| | passive | tultiin | $\text{-t}_{\text{PASSIVE}}\text{-i}_{\text{TAM}}\text{-Vn}_{\text{AGR}}$ |
| conditional mood | 1st sing. | tulisin | $\text{-isi}_{\text{TAM}}\text{-n}_{\text{AGR}}$ |
| (-isi-, [Karlsson, 1999, Section 65]) | 2nd sing. | tulisit | $\text{-isi}_{\text{TAM}}\text{-t}_{\text{AGR}}$ |
| | 3rd sing. | tulisi | $\text{-isi}_{\text{TAM}}\text{-V}_{\text{AGR}}$ |
| | 1st plur. | tulisimme | $\text{-isi}_{\text{TAM}}\text{-mme}_{\text{AGR}}$ |
| | 2nd plur. | tulisitte | $\text{-isi}_{\text{TAM}}\text{-tte}_{\text{AGR}}$ |
| | 3rd plur. | tulisivat | $\text{-isi}_{\text{TAM}}\text{-vat}_{\text{AGR}}$ |
| | passive | tultaisiin | $\text{-ta}_{\text{PASSIVE}}\text{-isi}_{\text{TAM}}\text{-Vn}_{\text{AGR}}$ |
| imperative mood | 1st sing. | – | |
| [Karlsson, 1999, Section 66] | 2nd sing. | tule | - |
| | 3rd sing. | tulkoon | $\text{-kV}_{\text{TAM}}\text{-oon}_{\text{AGR}}$ |
| | 1st plur. | tulkaamme | $\text{-kV}_{\text{TAM}}\text{-mme}_{\text{AGR}}$ |
| | 2nd plur. | tulkaa | $\text{-kV}_{\text{TAM}}$ |
| | 3rd plur. | tulkoot | $\text{-kV}_{\text{TAM}}\text{-vat}_{\text{AGR}}$ |
| | passive | tultakoon | $\text{-ta}_{\text{PASSIVE}}\text{-kV}_{\text{TAM}}\text{-Vn}_{\text{AGR}}$ |
| potential mood | 1st sing. | tullen | $\text{-ne}_{\text{TAM}}\text{-n}_{\text{AGR}}$ |
| (-ne-, [Karlsson, 1999, Section 67]) | 2nd sing. | tullet | $\text{-ne}_{\text{TAM}}\text{-t}_{\text{AGR}}$ |
| | 3rd sing. | tullee | $\text{-ne}_{\text{TAM}}\text{-V}_{\text{AGR}}$ |
| | 1st plur. | tullemme | $\text{-ne}_{\text{TAM}}\text{-mme}_{\text{AGR}}$ |
| | 2nd plur. | tullette | $\text{-ne}_{\text{TAM}}\text{-tte}_{\text{AGR}}$ |
| | 3rd plur. | tullevat | $\text{-ne}_{\text{TAM}}\text{-vat}_{\text{AGR}}$ |
| | passive | tultaneen | $\text{-t}_{\text{PASSIVE}}\text{-ne}_{\text{TAM}}\text{-Vn}_{\text{AGR}}$ |

Table S7: Selected forms of Finnish verbs. See text for details.

| Slot | Label | Function | Morphs | Reference |
|---|---|---|---|---|
| Valence | TIR$_1$ | Causative | -tIr | |
| Voice | IL$_2$ | Passive | -Il | |
| Polarity | MA$_3$ | Negation | -mA | |
| Mood | EBIL$_4$ | Abilitative | -Ebil | van Schaaik [2020], section 21 |
| | IN$_4$ | Imperative | -In | van Schaaik [2020], section 18.1 |
| TAM1 | AR$_5$ | Aorist | -(I/E)r, -z | van Schaaik [2020], section 20.5 ('Present-2') |
| | IYOR$_5$ | Imperfective | -Iyor | |
| | ACAK$_5$ | Future | -AcAk | van Schaaik [2020], section 20.2 |
| | MIS1$_5$ | Perfective | -mIş | van Schaaik [2020], section 20.3 |
| | TI$_5$ | Past | -dI | van Schaaik [2020], section 20.4 |
| 3rd Plural | lar$_6$ | 3rd Plural | -lAr | |
| TAM2 | MUS2$_7$ | Aorist | -mIş | van Schaaik [2020], section 24.2 |
| | DU$_7$ | Past | -dI | van Schaaik [2020], section 24.1.3 |
| Agreement | IM$_8$ | 1st singular | -Im | |
| | SIN$_8$ | 2nd singular | -sIn | |
| | IZ$_8$ | 1st plural | -Iz | |
| | SINIZ$_8$ | 2nd plural | -sInIz | |
| | K$_8$ | 1st plural, past direct | -k | |

Table S8: Verb suffixes in Turkish, arranged by slot. The first column describes slots as in the main paper. The second column lists the unique identifiers for each morpheme. The third column describes the function and meaning; the fourth column describes the approximate phonological form (see van Schaaik [2020, Section 5.1] for the vowels I, A, E).

| | | 1 sg. | 2 sg. | 3 sg. | 1 pl. | 2 pl. | 3 pl. |
|---|---|---|---|---|---|---|---|
| Indicative Non-Past | Aorist | yaparım<br>-AR$_5$-IM$_8$ | yaparsın<br>-AR$_5$-SIN$_8$ | yapar<br>-AR$_5$ | yaparız<br>-AR$_5$-IZ$_8$ | yaparsınız<br>-AR$_5$-SINIZ$_8$ | yaparlar<br>-AR$_5$-LAR$_6$ |
| | Imperfective | yapıyorum<br>-IYOR$_5$-IM$_8$ | yapıyorsun<br>-IYOR$_5$-SIN$_8$ | yapıyor<br>-IYOR$_5$ | yapıyoruz<br>-IYOR$_5$-IZ$_8$ | yapıyorsunuz<br>-IYOR$_5$-SINIZ$_8$ | yapıyorlar<br>-IYOR$_5$-LAR$_6$ |
| | Future | yapacağım<br>-ACAK$_5$-IM$_8$ | yapacaksın<br>-ACAK$_5$-SIN$_8$ | yapacak<br>-ACAK$_5$ | yapacağız<br>-ACAK$_5$-IZ$_8$ | yapacaksınız<br>-ACAK$_5$-SINIZ$_8$ | yapacaklar<br>-ACAK$_5$-LAR$_6$ |
| Indicative Past | Perfective | yaptım<br>-TI$_5$-IM$_8$ | yaptın<br>-TI$_5$-SIN$_8$ | yaptı<br>-TI$_5$ | yaptık<br>-TI$_5$-K$_8$ | yaptınız<br>-TI$_5$-SINIZ$_8$ | yaptılar<br>-TI$_5$-LAR$_6$ |
| | Imperfective | yapıyordum<br>-IYOR$_5$-DU$_7$-IM$_8$ | yapıyordun<br>-IYOR$_5$-DU$_7$-SIN$_8$ | yapıyordu<br>-IYOR$_5$-DU$_7$ | yapıyorduk<br>-IYOR$_5$-DU$_7$-K$_8$ | yapıyordunuz<br>-IYOR$_5$-DU$_7$-SINIZ$_8$ | yapıyorlardı<br>-IYOR$_5$-LAR$_6$-DU$_7$ |
| Non-Indicative | Perfective | yapmışım<br>-MIS$_5$-IM$_8$ | yapmışsın<br>-MIS$_5$-SIN$_8$ | yapmış<br>-MIS$_5$ | yapmışız<br>-MIS$_5$-IZ$_8$ | yapmışsınız<br>-MIS$_5$-SINIZ$_8$ | yapmışlar<br>-MIS$_5$-LAR$_6$ |
| | Aorist | yaparmışım<br>-AR$_5$-MIS$_7$-IM$_8$ | yaparmışsın<br>-AR$_5$-MIS$_7$-SIN$_8$ | yaparmış<br>-AR$_5$-MIS$_7$ | yaparmışız<br>-AR$_5$-MIS$_7$-IZ$_8$ | yaparmışsınız<br>-AR$_5$-MIS$_7$-SINIZ$_8$ | yaparlarmış<br>-AR$_5$-LAR$_6$-MIS$_7$ |
| | Imperfective | yapıyormuşum<br>-IYOR$_5$-MIS$_7$-IM$_8$ | yapıyormuşsun<br>-IYOR$_5$-MIS$_7$-SIN$_8$ | yapıyormuş<br>-IYOR$_5$-MIS$_7$ | yapıyormuşuz<br>-IYOR$_5$-MIS$_7$-IZ$_8$ | yapıyormuşsunuz<br>-IYOR$_5$-MIS$_7$-SUNUZ$_8$ | yapıyorlarmuş<br>-IYOR$_5$-LAR$_6$-MIS$_7$ |

Table S9: Illustrating Turkish verb suffixes using selected forms of the verb *yapmak*, covering TAM and agreement suffixes (slots 5–8). Note that the labeling of TAM categories varies between grammars, and our choices here are only intended to provide a broad orientation. See text for details.

| Slot | Morpheme | Function | Reference |
|---|---|---|---|
| Valence | $\text{AT}_{\text{VAL}}$ | Causative | Rounds [2001] 4.5.3 |
| Mood | $\text{HET}_{\text{MOOD}}$ | Potential | Rounds [2001] 4.5.1 |
| | $\text{NE}_{\text{MOOD}}$ | conditional | Rounds [2001] 4.3.7 |
| | $\text{J}_{\text{MOOD}}$ | subjunctive | Rounds [2001] 4.3.5 |
| Tense | $\text{T}_{\text{TENSE}}$ | indicative past | Rounds [2001] 4.3.3 |
| Agreement (Obj+Subj) | | See Table S11 | Rounds [2001] 4.3 |

Table S10: Hungarian verb suffixes

In addition to finite verbs, infinitives are also labeled as verbs in the corpus. These have an ending *-ni* [Rounds, 2001, 4.4.4]; we assign it a slot "Nominal" for our analysis..

## S1.5 Sesotho

We describe Sesotho verb affixes following Hahn et al. [2021]. who described these based on [Doke and Mofokeng, 1967, Guma, 1971, Demuth, 1992]. We refer to Hahn et al. [2021] for further details.

1. Subject agreement: This morpheme encodes agreement with the subject, for person, number, and noun class (the latter only in the 3rd person) [Doke and Mofokeng, 1967, §395]. The annotation provided by Demuth [1992] distinguishes between ordinary subject agreement prefixes and agreement prefixes used in relative clauses; we distinguish these morpheme types here.

2. Negation [Doke and Mofokeng, 1967, §429]

3. Tense/aspect marker [Doke and Mofokeng, 1967, §400–424]

4. Object agreement or reflexive marker [Doke and Mofokeng, 1967, §459]. Similar to subject agreement, object agreement denotes person, number, and noun class features of the object.

We identified the following suffixes:

1. Semantic derivation: reversive (e.g., 'do' → 'undo')

2. Valence: (e.g., causative, neuter/stative, applicative, and reciprocal)

3. Voice: passive

4. Tense/Aspect

5. Mood

6. Relative marker, marking verbs in relative clauses

## S1.6 Japanese

We describe Japanese verb suffixes following [Hahn et al., 2021], who described these based on [Kaiser et al., 2013, Hasegawa, 2014]. We refer to [Hahn et al., 2021] for details.

| | | | Singular | | | Plural | | | Rounds [2001] |
|---|---|---|---|---|---|---|---|---|---|
| | | | 1 | 2 | 3 | 1 | 2 | 3 | |
| Ind | Pres | I | teszek | teszel | tesz | teszünk | tesztek | tesznek | |
| | | | csinálok | csinálsz | csinál | csinálunk | csináltok | csinálnak | |
| | | | ismerek | ismersz | ismer | ismerünk | ismertek | ismernek | |
| | | | -ek | -sz | – | -ünk | -tek | -nek | 4.3.1.1 |
| | | | -$EK_{AGR}$ | -$EL_{AGR}$ | – | -$UNK_{AGR}$ | -$TEK_{AGR}$ | -$NEK_{AGR}$ | |
| | | D | teszem | teszed | teszi | tesszük | teszitek | teszik | |
| | | | csinálom | csinálod | csinálja | csináljuk | csináljátok | csinálják | |
| | | | ismerem | ismered | ismeri | ismerjük | ismeritek | ismerik | |
| | | | -em | -ed | -i | -jük | -itek | -ik | 4.3.1.2 |
| | | | -$EM_{AGR}$ | -$ED_{AGR}$ | -$I_{AGR}$ | -$IUK_{AGR}$ | -$ITEK_{AGR}$ | -$IK_{AGR}$ | |
| | Past | I | tettem | tettél | tett | tettünk | tettetek | tettek | |
| | | | csináltam | csináltál | csinált | csináltunk | csináltatok | csináltak | |
| | | | ismertem | ismertél | ismert | ismertünk | ismertetek | ismertek | |
| | | | -t-em | -t-él | -t | -t-ünk | -t-etek | -t-ek | 4.3.3.1 |
| | | | -$T_{TENSE}$-$EM_{AGR}$ | -$T_{TENSE}$-$EEL_{AGR}$ | -$T_{AGR}$ | -$T_{TENSE}$-$UNK_{AGR}$ | -$T_{TENSE}$-$ETEK_{AGR}$ | -$T_{TENSE}$-$EK_{AGR}$ | |
| | | D | tettem | tetted | tette | tettük | tettétek | tették | |
| | | | csináltam | csináltad | csinálta | csináltuk | csináltátok | csinálták | |
| | | | ismertem | ismerted | ismerte | ismertük | ismertétek | ismerték | |
| | | | -t-em | -t-ed | -t-e | -t-ük | -t-étek | -t-ék | 4.3.3.2 |
| | | | -$T_{TENSE}$-$EM_{AGR}$ | -$T_{TENSE}$-$ED_{AGR}$ | -$T_{TENSE}$-$I_{AGR}$ | -$T_{TENSE}$-$UK_{AGR}$ | -$T_{TENSE}$-$EETEK_{AGR}$ | -$T_{TENSE}$-$EEK_{AGR}$ | |
| Cond | Pres | I | tennék | tennél | tenne | tennénk | tennétek | tennének | |
| | | | csinálnék | csinálnál | csinálna | csinálnánk | csinálnátok | csinálnának | |
| | | | ismernék | ismernél | ismerne | ismernénk | ismernétek | ismernének | |
| | | | -nék | -nél | -ne | -nénk | -nétek | -nének | 4.3.7.1 |
| | | I | -$NE_{MOOD}$-$EK_{AGR}$ | -$NE_{MOOD}$-$EL_{AGR}$ | -$NE_{MOOD}$ | -$NE_{MOOD}$-$ENK_{AGR}$ | -$NE_{MOOD}$-$ETEK_{AGR}$ | -$NE_{MOOD}$-$ENEK_{AGR}$ | |
| | | D | tenném | tennéd | tenné | tennénk | tennétek | tennék | |
| | | | csinálnám | csinálnád | csinálná | csinálnánk | csinálnátok | csinálnák | |
| | | | ismerném | ismernéd | ismerné | ismernénk | ismernétek | ismernék | |
| | | | -ném | -néd | -né | -nénk | -nétek | -nék | 4.3.7.2 |
| | | | -$NE_{MOOD}$-$EM_{AGR}$ | -$NE_{MOOD}$-$ED_{AGR}$ | -$NE_{MOOD}$-$E_{AGR}$ | -$NE_{MOOD}$-$ENK_{AGR}$ | -$NE_{MOOD}$-$ETEK_{AGR}$ | -$NE_{MOOD}$-$EK_{AGR}$ | |
| Subj | Pres | I | tegyek | tégy or tegyél | tegyen | tegyünk | tegyetek | tegyenek | |
| | | | csináljak | csinálj or csináljál | csináljon | csináljunk | csináljatok | csináljanak | |
| | | | ismerjek | ismerj or ismerjél | ismerjen | ismerjünk | ismerjetek | ismerjenek | |
| | | | -j-ek | -j-(él) | -j-en | -j-ünk | -j-etek | -j-enek | 4.3.5.1 |
| | | | -$J_{MOOD}$-$EK_{AGR}$ | $J_{MOOD}$-$EEL_{AGR}$ | -$J_{MOOD}$-$EN_{AGR}$ | -$J_{MOOD}$-$UNK_{AGR}$ | -$J_{MOOD}$-$ETEK_{AGR}$ | -$J_{MOOD}$-$ENEK_{AGR}$ | |
| | | D | tegyem | tedd or tegyed | tegye | tegyük | tegyétek | tegyék | |
| | | | csináljam | csináld or csináljad | csinálja | csináljuk | csináljátok | csinálják | |
| | | | ismerjem | ismerd or ismerjed | ismerje | ismerjük | ismerjétek | ismerjék | |
| | | | -j-em | -j-ed | -j-e | -j-ük | -j-étek | -j-ék | 4.3.5.2 |
| | | | -$J_{MOOD}$-$EM_{AGR}$ | -$J_{MOOD}$-$ED_{AGR}$ | -$J_{MOOD}$-$E_{AGR}$ | -$J_{MOOD}$-$UK_{AGR}$ | -$J_{MOOD}$-$EETEK_{AGR}$ | -$J_{MOOD}$-$EEK_{AGR}$ | |

Table S11: Hungarian verb suffixes (see text for details): Inflection by tense, mood, definitiness, and person/number. "Ind" = "Indicative", "Cond" = "Conditional", "Subj" = "Subjunctive", "I" = "Indefinite", "D" = "Definite". For each form, we show the orthographic shape for three verbs, the suffix as identified by Rounds [2001], and our representation as a string of morphemes as described in Table S10.

| | | |
|---|---|---|
| | Form | *juttuihisi* |
| | Lemma | *juttu* |
| FINNISH | Annotation | `Case=Ill\|Number=Plur\|Number[psor]=Sing\|Person[psor]=2` |
| | Segmentation | -I-HI-SI |
| | Gloss | -PLURAL-ILLATIVE-2SGPOSS |
| | Form | *érveiket* |
| | Lemma | *érv* |
| HUNGARIAN | Annotation | `Case=Acc\|Number=Plur\|Number[psor]=Plur\|Person[psor]=3` |
| | Segmentation | -EK-K-ET |
| | Gloss | -PLURAL-POSSESSOR3RDPLUR-ACCUSATIVE |
| | Form | *hareketlerimden* |
| | Lemma | *hareket* |
| TURKISH | Annotation | `Case=Abl\|Number=Plur\|Number[psor]=Sing\|Person=3\|Person[psor]=1` |
| | Segmentation | -LAR-IM-DEN |
| | Gloss | PLURAL-1SGPOSS-ABLATIVE |

Table S12: Noun affixes in Finnish, Hungarian, and Turkish.

1. *suru*: obligatory suffix after Sino-Japanese words when they are used as verbs
2. Valence: causative (-*ase*-)
3. Voice and Mood: passive (-*are*-, -*rare*-) and potential (-*e*-, -*are*-, -*rare*-)
4. Mood: desiderative (-*ta*-)
5. Politeness (-*mas*-)
6. Polarity (Negation -*n*-)
7. Tense, Aspect, Mood: past (-*ta*), future/hortative (-*yoo*) [Kaiser et al., 2013, 229]
8. (Non-)Finiteness (-*te*)

### S1.7 Nouns in Finnish. Hungarian, and Turkish

We illustrate noun suffixes using examples taken from the corpora, with the annotation given in UD and our segmentation, in Table S12. In addition to these three slots, Hungarian also has possessed suffixes, marked with `Number[psed]` in the data. However, this is rare in the data, occurring only three times in the training set, and was thus excluded.

## S2 Other Measures of Accuracy

Here we report results using two alternative measures of accuracy. FULLACCURACY measures the fraction of forms (with at least two affixes) in the corpus that are ordered entirely correctly (Figure S1). FULLTYPEACCURACY modifies this by disregarding repeated occurrences of the same form in the corpus (Figure S2). For both measures, we only include forms that have at least two affixes; the other forms are ordered correctly under any ordering. Results across these two measures and the one reported in the main paper

strongly agree both numerically and in the qualitative patterns of the comparison between optimized orderings and baselines.

# S3 Technical Details

## S3.1 Computation of Memory-Surprisal Tradeoff

As described in the main paper, we treat word forms as sequences $w_1 \dots w_L$ of morphemes. The basic ingredient to estimating the bound on the memory-surprisal tradeoff curve is the conditional probability of a morpheme $w_t$ given $t$ preceding morphemes within a word token:

$$P_t(w_k | w_{k-t} \dots w_{k-1}) \tag{1}$$

$P_t$ is defined to only depend on the relative sequence of the morphemes $w_{k-t} \dots w_{k-1} w_k$, not on the absolute value of $k \in \{1, \dots, L\}$. That is, it does not keep track of the absolute position of the morphemes in a word.

The conditional distribution $P_t$ is defined for every $t = 0, 1, \dots$; it formally defines a $(t+1)$-th order Markov model. When $w_{k-t} \dots w_{k-1}$ extends across the beginning of the word (that is, if $k - t < 1$), $P_t$ is conditioned simply on the entire prefix of length $k - 1 < t$. To ensure that $P_t$ defines a proper normalized distribution over word forms (i.e., finite sequences of morphemes), we prefix and suffix morpheme sequences with special start-of-word and end-of-word symbols, respectively, in the definition of $P_t$.

Then, by combining Equations (6) and (7) from the main paper and plugging in the observed corpus distribution for $P$, $I_t$ (Equation 5 in the main paper) can be written as

$$I_t \equiv \mathrm{I}[w_t : w_0 | w_1 \dots w_{t-1}] \equiv \sum_{w_0 \dots w_t} \frac{F(w_0 \dots w_t)}{|C|} \log_2 \frac{P_t(w_t | w_0 \dots w_{t-1})}{P_{t-1}(w_t | w_1 \dots w_{t-1})}. \tag{2}$$

where the sum runs over all word tokens $w_1 \dots w_L$ in the corpus and all morphemes $w_t$ within those; $F(w_0 \dots w_t) \in \{0, 1, 2, \dots\}$ is the frequency of the morpheme sequence $w_0 \dots w_t$ in the corpus, and $|C|$ is the total number of morphemes in the corpus. Evaluating Equations (6) and (7) from the main paper for every $T = 1, 2, \dots$, one then obtains a bound on the memory-surprisal tradeoff curve.

Following Hahn et al. [2021], we use Kneser-Ney smoothing to estimate $P_t$. Further following Hahn et al. [2021], disjoint sets of data are used for estimating $P(w_t | w_0 \dots w_{t-1})$ and $F(w_0 \dots w_t)$: All datasets were split into *training* and *held-out* partitions. For the Universal Dependencies corpora, these were already split (we used the *validation* partitions for held-out). For Sesotho, we used the split of Hahn et al. [2021]. The heldout partition consisted of between 5% (Sesotho) and 43% (Hungarian verbs) of all tokens. We use the word form tokens in the training partition to estimate $P_t$, and then use the word form tokens in the held-out partition to estimate $F$ (2). Estimating mutual information using two separate datasets in this way is a common approach with theoretical guarantees [McAllester and Stratos, 2020] that prevents an overestimation bias arising when using a single dataset.

For each language, we parameterize possible morpheme orderings through the $N!$ possible orderings of the $N$ affix slots. Applying any such ordering to the forms extracted from the corpus results in a set of counterfactual forms with some associated memory-surprisal tradeoff curve.

**Optimization Method** Further following Hahn et al. [2021], we optimize orderings using an adaptation of the hill-climbing method originally devised by Gildea and Temperley [2007] for optimizing word order. The algorithm randomly initializes the ordering of slots. It then iteratively changes it to decrease AUC. In every

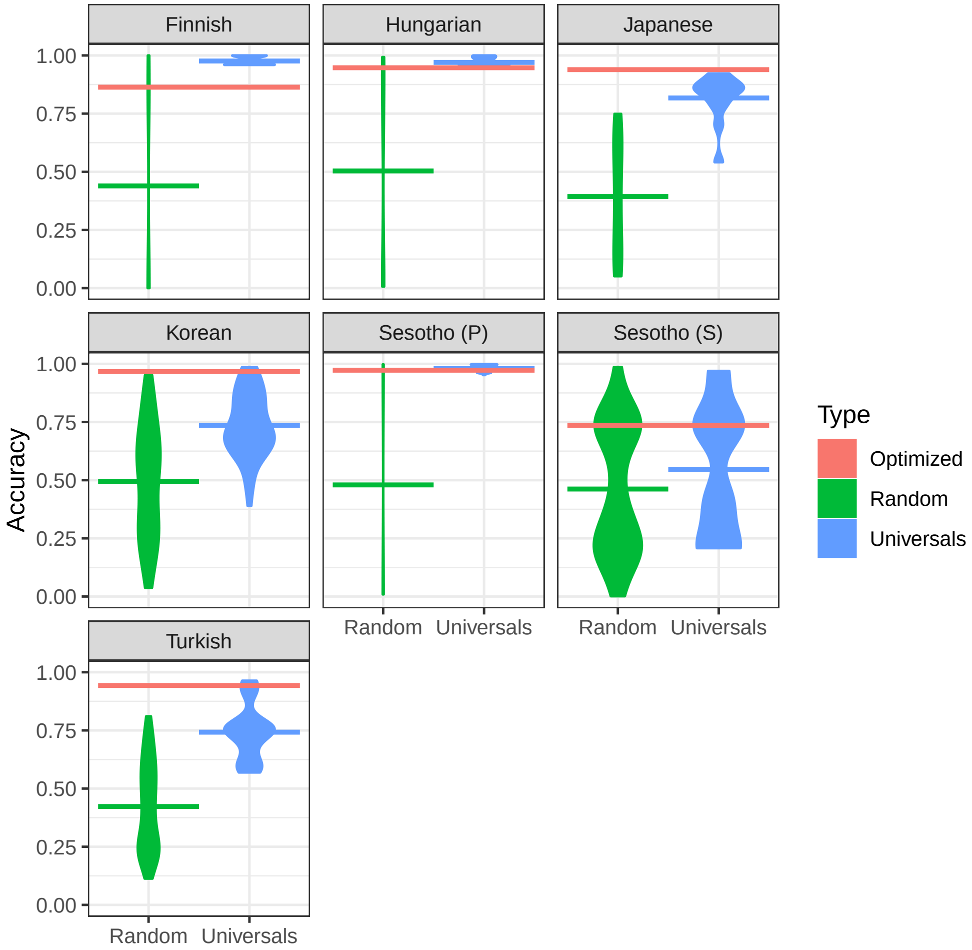


Figure S1: Accuracies in predicting verb morpheme ordering, according to the FULLACCURACY measure. For the baselines, we provide both a smoothed violin plot of the distribution of accuracies, and the mean accuracy as a horizontal line (green and blue). For the optimized order, we show the accuracy as a horizontal line (red). In all languages, optimized orderings provide higher accuracy than the mean of the random baselines at $p < 0.001$.

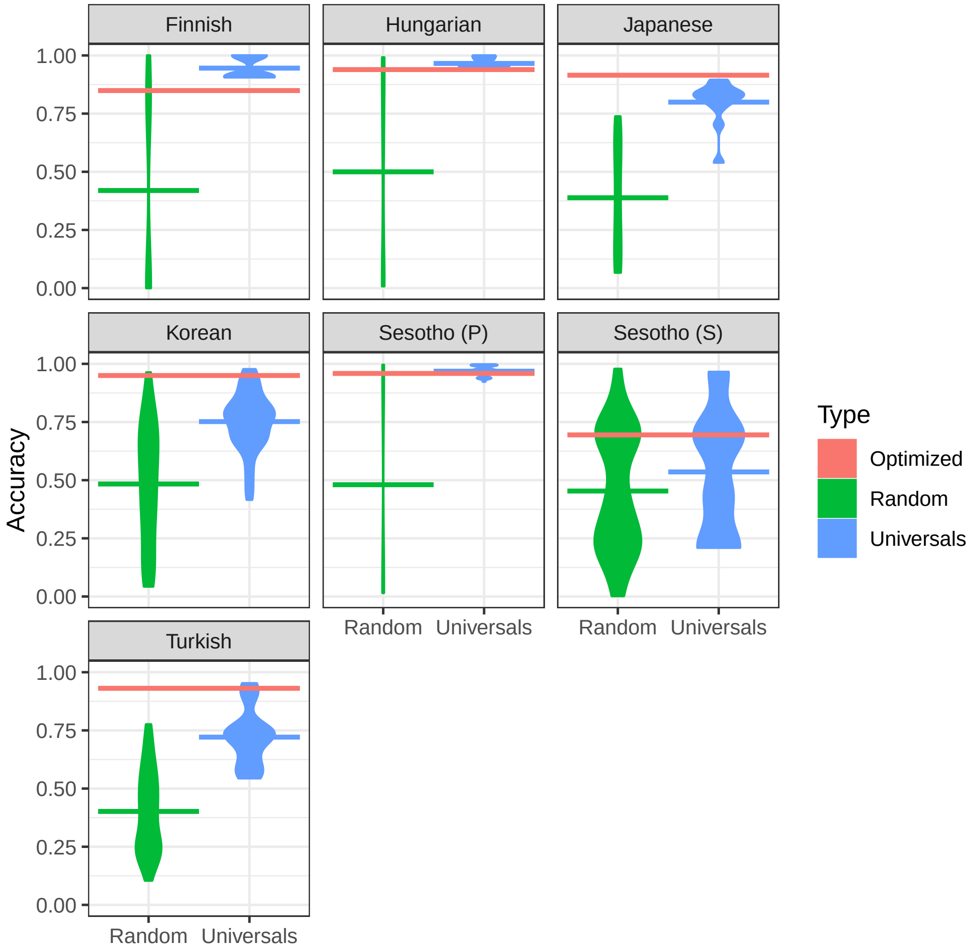


Figure S2: Accuracies in predicting verb morpheme ordering, according to the FULLTYPEACCURACY measure. For the baselines, we provide both a smoothed violin plot of the distribution of accuracies, and the mean accuracy as a horizontal line (green and blue). For the optimized order, we show the accuracy as a horizontal line (red). In all languages, optimized orderings provide higher accuracy than the mean of the random baselines at $p < 0.001$.

iteration, it randomly chooses a slot, and moves it to some other position (between two other slots, or at the beginning or end) in such a way to maximally decrease AUC. To speed up computation, slots occurring fewer than 10 times are sampled only in about 5% of iterations, and the algorithm considers only a random subset of about half of all possible positions in every iteration. Optimization runs for 1,000 iterations, empirically sufficient to guarantee convergence. Due to the difficulty of combinatorial optimization, this algorithm is not guaranteed to find the global optimum. However, as Gildea and Temperley [2007] previously, we find its results to be consistent across runs and to have lower AUC than all randomly sampled orderings.

**On the Role of Conditional Mutual Information** We note that some prior work has considered the role of unconditional mutual information $I[w_t : w_0]$ in human language [e.g. Ebeling and Pöschel, 1994, Futrell et al., 2020], whereas the bounds used here are based on conditional mutual information. Informally, the choice of conditional mutual information reflects the fact that utilizing information from the distant past induces memory load only to the extent that it is not redundantly reflected in the intervening material; conditional mutual information only reflects those bits of predictive information that cannot be redundantly inferred from intervening material. Corresponding theoretical bounds on memory load cannot be obtained in general considering only unconditional mutual information [Hahn et al., 2021].

## S3.2 Example Computation of Memory-Surprisal Tradeoff

Here, we provide an example for the calculation of the bound on the memory-surprisal tradeoff curve and its AUC, using the example of Sesotho verb suffixes, comparing real and reverse orderings.

**Conditional Mutual Information** Recall that the conditional mutual information $I_t$ is defined as (Equatuion 2):

$$I_t \equiv \mathrm{I}[w_t : w_0 | w_1 \dots w_{t-1}] \equiv \sum_{w_0 \dots w_t} \frac{F(w_0 \dots w_t)}{|C|} \log_2 \frac{P_t(w_t | w_0 \dots w_{t-1})}{P_{t-1}(w_t | w_1 \dots w_{t-1})}. \tag{3}$$

The resulting curve for Sesotho suffixes is shown in Figure S3 (bottom left). There is a difference, though barely perceptible, whereby $I_t$ decays faster for the real orderings than for the reverse orderings.

To understand whether this difference is meaningful, it is helpful to consider $I_t$ for individual suffix classes. In Figure S3 (top), we show this quantity separately for the different types of suffixes in Sesotho, that is, we restrict the summation $\sum_{w_0 \dots w_t}$ and the normalization constant $|C|$ to those sequences where $w_t$ is an affix of a specific morphological category. The results show that, for several of the affix classes, the decay of $I_t$ is distinctively steeper for the real ordering than the reverse ordering. For instance, for the Derivation affix class, conditional mutual information with the immediately preceding morpheme is substantially higher in the real orderings (where the preceding morpheme is the root) than in reverse orderings (where the preceding morpheme is an inflectional morpheme). As described in the main paper, such a steep fall-off indicates that predictive information is concentrated in the recent past, which reduces the memory load needed for accurate prediction. Importantly, no affix class shows a clear pattern in the reverse direction.

By averaging these curves, weighted by the corpus frequency of different affix types, one obtains the overall curves for $I_t$ shown in Figure S3 (bottom left). The resulting curves mirror the pattern observed in the individual curves, though the numerical difference between real and reverse orderings is small: This is a common phenomenon when calculating memory-surprisal tradeoffs: It turns out that the Mood affix is by far the most common one, and thus dominates the overall average information. In general, high-frequency morphemes will tend to be less sensitive to ordering differences, because their high frequency makes them

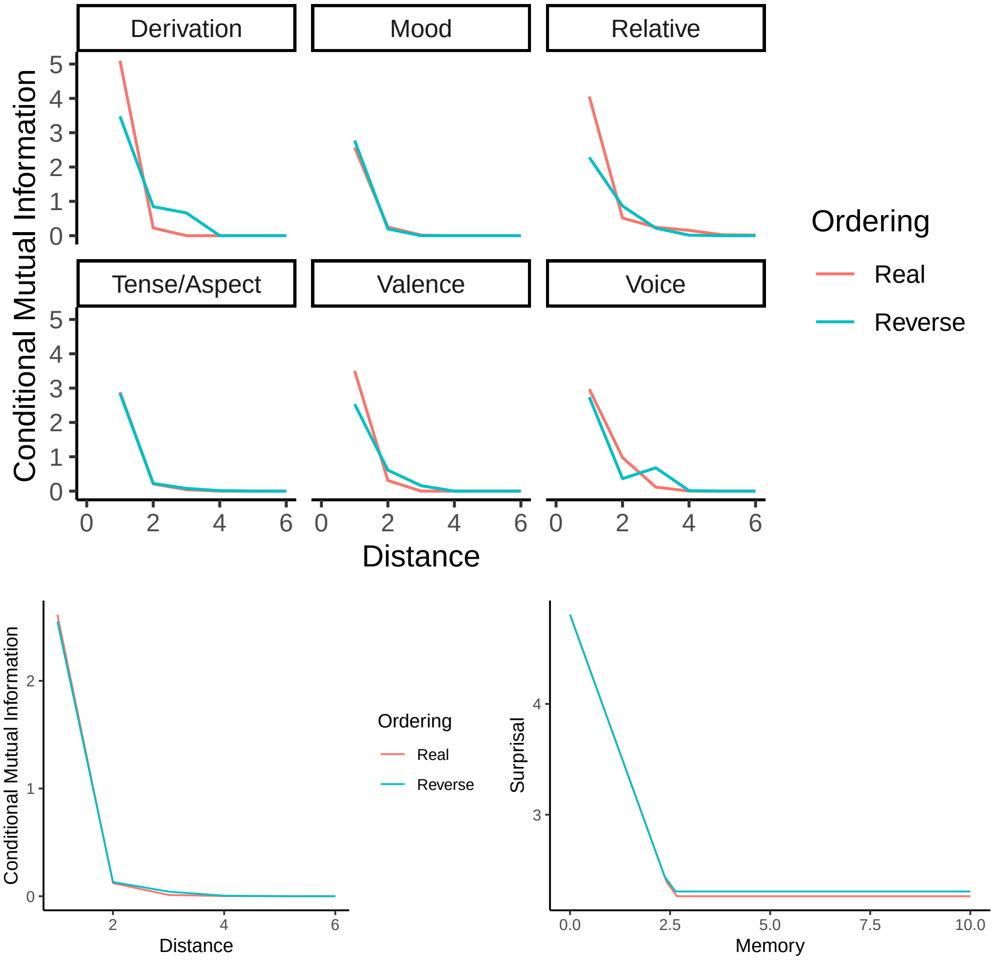


Figure S3: Conditional mutual information $I_t$ (top and bottom left) and memory-surprisal tradeoff (bottom right) for Sesotho verb suffixes. See text for details.

always relatively easy to predict. Differences between the efficiency of different orderings are thus typically driven by lower-frequency affixes for which ordering strongly impacts how easy they are to predict from their immediate context. Considering affix classes individually shows that the overall difference, even though small, is meaningful.

**Tradeoff Curve** By further applying Equations (6–7) from the main paper and tracing out all values of $T = 1, 2, 3, \ldots$, one then obtains a bound on the memory-surprisal tradeoff curves, shown in Figure S3 (bottom right). More specifically, for every $T = 1, 2, 3, \ldots$, one computes memory capacity as

$$M_T := \sum_{t=1}^{T} t I_t. \tag{4}$$

and surprisal as

$$S_T := S_\infty + \sum_{t=T+1}^{\infty} I_t. \tag{5}$$

By connecting these points, one obtains a bound on the tradeoff curve as shown in Figure S3 (bottom right). Due to the factor $t$ inside $M_T$, a steeper fall-off in $I_t$ translates into lower memory capacity $M_T$ needed for the same level of surprisal $S_T$. For some sufficiently large $T_{max}$ (bounded by the length of the longest word form in the corpus), $I_{T_{max}}$ will become zero, and $M_T$ and $S_T$ will not change any more as $T$ increases beyond $T_{max}$. In this case, $S_{T_{max}}$ will equal $S_\infty$, the surprisal obtained with perfect memory. In the case of Figure S3 (bottom left), this situation is attained at $T_{max} = 4$. For any comprehender with memory capacity greater than $M_{T_{max}}$, surprisal will still be at least $S_{T_{max}} = S_\infty$; thus, the tradeoff curve in theory extends infinitely to the right horizontally (we show it up to $M = 10$ in the figure).

These curves reflect the Pareto frontiers for possible comprehenders: For any comprehender, its average surprisal and average memory usage cannot lie below the curve. The real orderings have a slightly more efficient tradeoff than the reverse orderings, as surprisal is lower at high memory capacities. Again, the numerical difference between real and reverse orderings appears small, but we note that this difference applies, on average, at every single morpheme in every word. A small change in the per-morpheme surprisal can lead to more significant change in overall surprisal across a word form or a sentence. Specifically, while the average difference is small, considering $I_t$ separately for affix classes (Figure S3, top) shows that substantial surprisal differences should be expected on derivational affixes, relative clause markers, and valence and voice affixes.

**AUC** The AUC measure used for determining the overall efficiency of an ordering is then derived by calculating the area under the tradeoff curve; this is smaller when the tradeoff curve decays faster. As the tradeoff curve in theory extends infinitely to the right, AUC needs to be restricted to some arbitrary upper bound $M_{max}$ on memory, for which we follow Hahn et al. [2021] in taking $M_{max} = 10$ as in Figure S3 (bottom right) across all languages. Note that, while the choice of $M_{max}$ substantially impacts the *absolute value* of AUC, impact on the *relative efficiencies* of different orderings is negligible. Geometrically, a smaller AUC indicates that the set below the Pareto frontier, the set of "impossible comprehenders", is smaller, indicating a more efficient tradeoff.

**Efficiency and $S_\infty$** In the specific case of Figure S3, not only the tradeoff's efficiency, but also the overall surprisal $S_\infty$ differs between the two orderings even at unbounded memory, though this does not always have to be the case [see Hahn et al., 2021, for more discussion]. Figure S3 shows that, for several suffix types, the

real and reverse orderings contain similar amounts of overall predictive information about the affix, but the real ordering places it at a smaller distance, which creates a more efficient tradeoff.

## References


Jayeol Chun, Na-Rae Han, Jena D. Hwang, and Jinho D. Choi. Building universal dependency treebanks in korean. In *Proceedings of the Eleventh International Conference on Language Resources and Evaluation (LREC 2018)*, 2018.

Katherine Demuth. Acquisition of Sesotho. In *The cross-linguistic study of language acquisition*, pages 557–638. Lawrence Erlbaum Associates, 1992.

Clement Martyn Doke and S Machabe Mofokeng. *Textbook of southern Sotho grammar*. Longmans, 1967.

W Ebeling and T Pöschel. Entropy and Long-Range Correlations in Literary English. *Europhysics Letters (EPL)*, 26(4):241–246, May 1994. ISSN 0295-5075, 1286-4854. doi: 10.1209/0295-5075/26/4/001. URL http://stacks.iop.org/0295-5075/26/i=4/a=001?key=crossref.4a8da3b3f5e80b828e6995b6bfc3e5be.

Richard Futrell, Edward Gibson, and Roger P Levy. Lossy-context surprisal: An information-theoretic model of memory effects in sentence processing. *Cognitive science*, 44(3), 2020.

Daniel Gildea and David Temperley. Optimizing Grammars for Minimum Dependency Length. In *Proceedings of the 45th Annual Meeting of the Association of Computational Linguistics*, pages 184–191, Prague, Czech Republic, June 2007. Association for Computational Linguistics. URL http://www.aclweb.org/anthology/P07-1024.

Samson Mbizo Guma. *An outline structure of Southern Sotho*. Shuter and Shooter, 1971.

Michael Hahn, Judith Degen, and Richard Futrell. Modeling word and morpheme order in natural language as an efficient tradeoff of memory and surprisal. *Psychological Review*, 2021.

Yoko Hasegawa. *Japanese: A linguistic introduction*. Cambridge University Press, 2014.

Stefan Kaiser, Yasuko Ichikawa, Noriko Kobayashi, and Hilofumi Yamamoto. *Japanese: A comprehensive grammar*. Routledge, 2013.

Fred Karlsson. *Finnish: An Essential Grammar*. 1999.

David McAllester and Karl Stratos. Formal limitations on the measurement of mutual information. 108: 875–884, 2020. URL http://proceedings.mlr.press/v108/mcallester20a.html.

Carol Howe Rounds. *Hungarian: An Essential Grammar*. 2001.

Gerjan van Schaaik. *The Oxford Turkish Grammar*. Oxford University Press, 2020.

Jaehoon Yeon and Lucien Brown. *Korean: A Comprehensive Grammar*. 2010.
